# A 4-cyano-3-methylisoquinoline inhibitor of *Plasmodium falciparum* growth targets the sodium efflux pump PfATP4

**DOI:** 10.1038/s41598-019-46500-5

**Published:** 2019-07-16

**Authors:** Paul R. Gilson, Rasika Kumarasingha, Jennifer Thompson, Xinxin Zhang, Jocelyn Sietsma Penington, Robabeh Kalhor, Hayley E. Bullen, Adele M. Lehane, Madeline G. Dans, Tania F. de Koning-Ward, Jessica K. Holien, Tatiana P. Soares da Costa, Mark D. Hulett, Melissa J. Buskes, Brendan S. Crabb, Kiaran Kirk, Anthony T. Papenfuss, Alan F. Cowman, Belinda M. Abbott

**Affiliations:** 1 Burnet Institute, Melbourne, Victoria 3004 Australia; 2 Monash University, Melbourne, Victoria 3800 Australia; 3 La Trobe University, Melbourne, Victoria 3086 Australia; 4 The Walter and Eliza Hall Institute of Medical Research, Parkville, Victoria 3052 Australia; 5 Research School of Biology, Australian National University, Canberra, ACT 2601 Australia; 6 University of Melbourne, Melbourne, Victoria 3010 Australia; 7 St. Vincent’s Institute of Medical Research, Melbourne, Victoria 3065 Australia; 8 School of Medicine, Deakin University, Waurn Ponds, Victoria 3216 Australia

**Keywords:** Parasite biology, Molecular medicine

## Abstract

We developed a novel series of antimalarial compounds based on a 4-cyano-3-methylisoquinoline. Our lead compound MB14 achieved modest inhibition of the growth *in vitro* of the human malaria parasite, *Plasmodium falciparum*. To identify its biological target we selected for parasites resistant to MB14. Genome sequencing revealed that all resistant parasites bore a single point S374R mutation in the sodium (Na^+^) efflux transporter PfATP4. There are many compounds known to inhibit PfATP4 and some are under preclinical development. MB14 was shown to inhibit Na^+^ dependent ATPase activity in parasite membranes, consistent with the compound targeting PfATP4 directly. PfATP4 inhibitors cause swelling and lysis of infected erythrocytes, attributed to the accumulation of Na^+^ inside the intracellular parasites and the resultant parasite swelling. We show here that inhibitor-induced lysis of infected erythrocytes is dependent upon the parasite protein RhopH2, a component of the new permeability pathways that are induced by the parasite in the erythrocyte membrane. These pathways mediate the influx of Na^+^ into the infected erythrocyte and their suppression via RhopH2 knockdown limits the accumulation of Na^+^ within the parasite hence protecting the infected erythrocyte from lysis. This study reveals a role for the parasite-induced new permeability pathways in the mechanism of action of PfATP4 inhibitors.

## Introduction

Several species of *Plasmodium* parasites cause malaria disease in humans with *P. falciparum* responsible for the most deaths, estimated at 445,000 in 2016^1^. With more than 200 million clinical cases per year, malaria imposes enormous health, social and economic burdens upon the countries afflicted. Efforts to reduce malaria through the use of vector control programs, increased use of intermittent preventive drug treatment in pregnancy, and widespread availability of artemisinin combination therapies (ACTs) have achieved impressive results, reducing mortality by ~30% globally between 2010–2015^2^. However, this momentum is being threatened by the increasing spread of ACT resistant *P. falciparum* parasites in the greater Mekong region of south-east Asia^3^. With the possibility of ACT resistance spreading globally, particularly to Africa where the malaria burden is the greatest, there is an urgent need for the development and deployment of new antimalarial drugs with novel targets.

The brief period in the malaria parasite’s life-cycle in which the merozoite-stage parasite egresses from its host erythrocyte to infect another erythrocyte presents many novel drug targets. There are numerous parasite proteins critical for this process, some that are unique to the parasite and do not have orthologues in human cells^4^. One such potential parasite target is Apical membrane antigen 1 (AMA1), a merozoite surface protein that plays a role in anchoring the merozoite to the erythrocyte surface prior to invasion^5,6^. Antibodies that bind AMA1 can block the protein’s adhesive interactions, prevent invasion and halt parasite proliferation, which is why AMA1 has been extensively explored as a potential vaccine target^7–9^. AMA1 spans the merozoite’s plasma membrane and has a large receptor-binding ectodomain and a short C-terminal cytoplasmic domain (CPD)^5,10^. Recently we discovered that the AMA1 CPD is phosphorylated by *P. falciparum* cAMP-dependent protein kinase A (PfPKA) on serine 610 (S610) of the CPD, triggering an additional phosphorylation event on threonine 613 (T613) by glycogen synthase kinase 3 (GSK3)^11–13^. These phosphorylation events are necessary for efficient merozoite invasion, though the underlying mechanism remains unknown.

Compounds that inhibit PfPKA and PfGSK3, such as H89 and 5 v, respectively, not only block invasion but also impede blood stage growth with 50% effective concentration (EC_50_) values in the range 3 to 6 µM^12,14–16^. PfPKA is most strongly expressed late in the asexual blood stage and it phosphorylates many schizont and merozoite-stage proteins. The kinase is therefore probably important for a range of replication and invasion functions^17–19^.

As PfPKA is an attractive drug target we investigated the possibility of repurposing a 4-cyano-3-methylisoquinoline compound that had been shown previously to inhibit the activity of PKA from rat liver, with an IC_50_ of 0.04 µM against the catalytic subunit^20^. Human and PfPKA share about 50% identity and due to the vast evolutionary distance between *Plasmodium spp*. and humans there may be sufficient divergence between these PKAs to target PfPKA with minimal inhibition of human homologs^21^. This repurposing approach has been employed previously for parasite phosphodiesterase enzymes that degrade cAMP and cGMP^22^. These cyclic nucleotides, produced by adenylate and guanyl cyclases, respectively, stimulate PfPKA and PfPKG. Phosphodiesterases play a key role in cAMP and cGMP signalling pathways by degrading these nucleotides, thereby attenuating the signal and reducing kinase activity^22,23^.

Our lead 4-cyano-3-methylisoquinoline compounds inhibited the growth of chloroquine sensitive and resistant *P. falciparum* blood stage parasites with EC_50_ values of ~1 µM in 72 h growth assays^24^. Since we anticipated that the lead compounds might be targeting PfPKA we performed merozoite egress and invasion assays in the presence of the lead compounds. We found that egress was not blocked by the compounds but invasion was, with a 50% inhibitory concentration (IC_50_) value below 10 µM^24^. However, kinase activity assays with parasite-sourced PfPKA and exogenous cAMP indicated that none of our compounds inhibited PfPKA activity at low µM levels, unlike the commonly used PKA inhibitor H89^25^.

To gain insight into the parasite target of one of our lead compounds 3-methyl-1-(1-ethylpropylamino)isoquinoline-4-carbonitrile, also known as MB14 (or compound 25^24^), we selected for parasites resistant to MB14 and sequenced the genomes of these parasites. All of the MB14 resistant (MB14r) mutants shared a point mutation in PfATP4, a Na^+^ efflux pump that resides on the parasite’s plasma membrane^26,27^. PfATP4 serves to maintain Na^+^ homeostasis in the parasite cytoplasm by exporting Na^+^ ions, while at the same time importing H^+^ ions^26^. Like the PfATP4-targeting spiroindolones^28^, MB14 inhibited Na^+^-dependent ATPase activity in parasite membrane preparations, consistent with it targeting PfATP4 directly. MB14 resistant mutants, like other PfATP4 mutants, showed cross-resistance to the spiroindolone clinical candidate cipargamin, a potent PfATP4 inhibitor. Both MB14 and cipargamin induced lysis of infected erythrocytes, probably due to the osmotic swelling of the intracellular parasite as it accumulates Na^+^ following inhibition of PfATP4^29^. Here we present evidence for a critical role in this process of the RhopH2-regulated new permeability pathways induced by the parasite in the erythrocyte membrane. These pathways serve as the primary route of influx of Na^+^ into the infected cell and hence underpin the accumulation of Na^+^ by the intracellular parasite.

## Results

### MB14 resistant parasites all share a mutation in PfATP4

**Figure 1:**
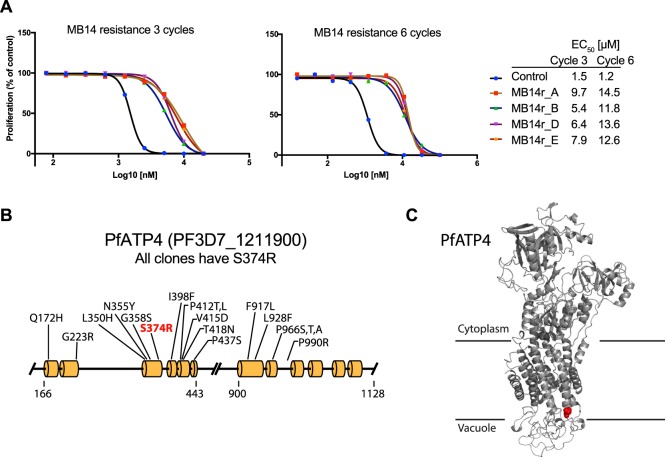
Blood stage *Plasmodium falciparum* parasites selected for resistance to the compound MB14 all share the same mutation in the gene for PfATP4. (**A**) After 3 and 6 cycles of selection on 10 µM of MB14, 72 h growth assays with a dilution series of MB14 were performed on the treated populations (A,B,D and E). Lactate dehydrogenase activity was measured and the EC_50_ for growth is shown. (**B**) Model of PfATP4 showing helical regions and the relative position of the S374R (red) mutation caused by MB14 selection is shown. Other mutations in PfATP4 that confer resistance to other inhibitors are indicated. This diagram is based on that of Jimenez-Diaz *et al*.^35^. (**C**) Structure of PfATP4 modelled on the human SERCA Ca^2+^ ATPase showing the predicted location of the S374R mutation with red spheres.

To select for resistance to MB14, the compound was added at 10 µM (~10 × EC_50_) to five populations of clonal 3D7 parasites (~1 × 10^8^ parasites). A control 3D7 population was treated with 0.1% v/v of the DMSO drug vehicle. The culture was monitored by microscopy and once most of the parasites had died the MB14 was removed and the cultures fed with MB14-free RPMI media. Once the population had recovered, MB14 treatment was resumed. A total of six treatment cycles were performed with the time to parasite death becoming longer and the recovery period becoming shorter. After cycles 3 and 6, the EC_50_ of MB14 was assessed by a 72 h growth assay using lactate dehydrogenase (LDH) activity to monitor parasite biomass^30^. After 3 cycles, the MB14 EC_50_ had increased several-fold and after 6 cycles it had increased even further (Fig. 1A). Resistance was selected in four out of five starting populations (A, B, D and E) with one population (C) never recovering after the first round of drug selection.

Several clonal lines derived from each resistant population were subsequently grown in the absence of MB14 for several weeks and then retested for MB14-sensitivity. In each case the parasites were found to maintain their resistance phenotypes (Fig. S1). Genomic DNA was prepared from the MB14 resistant (MB14r) clones as well as from clones of the untreated 3D7 control parasites. Genome sequences were derived from 18 cloned lines with good coverage ranging from 30- to 686-fold (mean ~200 fold) (Table S1). None of the clones showed large or consistent copy number alterations (gene amplifications) and the only rearrangements detected by the GRIDSS platform were in telomeric regions or in regions local to genes for erythrocyte membrane proteins^31^. Using two different small event callers (SNVer and VarScan2), single nucleotide polymorphisms (SNPs) that were not in the parental 3D7 clones were found in all clones of all four resistant lines (Table S1). One event, a T ->G mutation at Chr 12:531680, was present in all drug-resistant clones but not in untreated parasites (Fig. S2). Two more SNPs were present in all the MB14r_A clones (A5 and A6), but not in the other clones. There was also one SNP that occurred in only two out of four of the MB14r_ D clones (MB14r_D12 and MB14r_D14, Table S1). The presence or absence of these mutations in two 3D7 clones and MB14 resistant clones A5, A6, B7, B8, D11, D12, E15 and E16 was confirmed by amplifying 0.5 kb regions covering the mutations and directly sequencing the PCR products.

The T ->G mutation at Chr 12:531680 codes for a serine to arginine mutation at amino acid 374 (S374R) of the P-type ATPase PfATP4 (Fig. 1B,C) (PF3D7_1211900). PfATP4 functions to pump Na^+^ ions out of blood stage parasites in exchange for protons, thereby maintaining a low cytosolic [Na^+^] within the parasite, relative to the external environment^26^. PfATP4 is a ‘hot spot’ for growth-inhibitory compounds; 28 compounds from the 400 antimalarial compounds comprising the Medicine for Malaria Venture’s (MMV’s) Malaria Box^32^ and 11 compounds from the 125 antimalarial compounds in MMV’s Pathogen Box (https://www.pathogenbox.org) inhibit PfATP4^33,34^. Several promising therapeutic compounds that target PfATP4 are under preclinical development and some 40 mutations have been identified that confer resistance to various inhibitors of PfATP4 (Figs 1B and S3)^27,28,33,35–39^.

On the basis of a homology model of PfATP4, based on the structure of the human Sarco/Endoplasmic Reticulum Ca^2+^-ATPase (SERCA), the PfATP4 S374R mutation is predicted to be located near the tip of transmembrane spanning helix three, proximal to the parasitophorous vacuole space surrounding the intra-erythrocytic parasite (Fig. 1C)^28^. A computational prediction of the functional consequence of the S374R mutation for protein dynamics and stability, conducted using DynaMut^40^, predicts the S374R mutation to have a small, but significant (ΔΔG = 0.898 kcal/mol) stabilising effect on PfATP4. Whether the mutation falls within the binding site of MB14, thereby having a direct effect on the affinity of the interaction between the compound and the protein, or perhaps causes conformational changes to PfATP4 that alter MB14’s ability to bind somewhere else in the protein, has not been established.

### Mutations in other genes do not appear to enhance MB14 resistance

In addition to the S374R PfATP4 mutation, all the MB14r_A parasite clones had two other mutations. The first of these mutations encoded an A568T change in the putative anion exchanger PfSulP (PF3D7_1471200) on chromosome 14 (Figs S2, S4 and S5). This 664 amino acid (aa) protein is predicted to have 11–12 transmembrane helices and to localise to the parasite’s plasma membrane^41^. The A568T mutation is in a predicted cytoplasm-facing C-terminal domain. This region of the protein has homology to the sulfate transporter and anti-Sigma factor antagonist (STAS) domain that may be involved in regulating the trafficking and function of the transporter^41,42^.

The second mutation common to all the MB14r_A clones was a L942F mutation in PF3D7_1119900, a large 1749 aa *Plasmodium* protein encoded on chromosome 11 (Figs S2, S4 and S6). This mutation is also found towards the C-terminus of the protein, adjacent to a domain predicted to be predominately β-sheet. The only homology predicted by PFAM for PF3D7_1119900 to proteins of known function is to the Sec. 23-binding domain of Sec. 16 (PF12931), raising the possibility that PF3D7_1119900 might be involved in regulating Sec. 23-COPII vesicular transport.

In two of four clonal parasite lines of the MB14r_D parasites (D12 and D14) a mutation was found in the PfSec. 24 A protein (940 aa) encoded on chromosome 13. This protein is involved in COPII-mediated vesicular transport and the mutation (P795L) lies in the C-terminal, gelsolin-like domain of Sec. 23/24 (PFAM PF00626) (Figs S2, S4 and S7).

**Figure 2:**
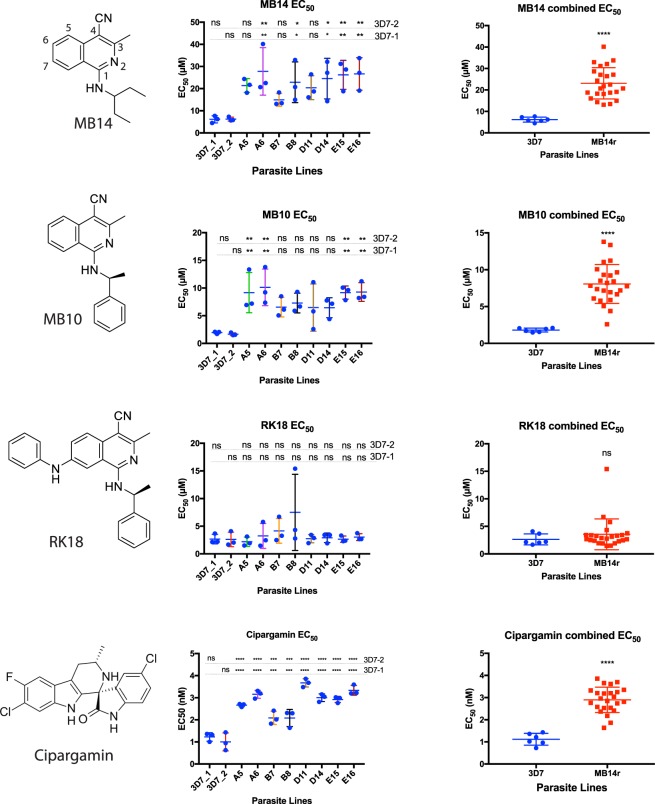
Blood stage MB14 resistant parasite clones are less sensitive than their parents to a compound structurally related to MB14, MB10, and the unrelated PfATP4 inhibitor cipargamin. Clonal wild-type 3D7 and MB14 resistant parasites were treated with a dilution series of the compounds indicated on the left and were grown for 72 h. (Middle) Parasite proliferation was measured using a SYBR green assay and the mean EC_50_ ± SD for growth indicated by bar and whiskers of three biological replicates (each of three technical replicates). The significance of differences between the mean EC_50_ of the resistant lines compared to each clonal 3D7 line is indicated where ns is non-significant, *p < 0.05, **p < 0.01, *** p < 0.001 and ****p < 0.0001; Ordinary one way ANOVA with Dunnett’s multiple comparisons test. (Right) Bar (mean) and whisker (±SD) scatter plots of the combined 3D7 (n = 6) mean EC_50_ versus the combined EC_50_ for MB14 resistant parasites (n = 24; Mann-Whitney T-test).

To determine whether the mutations additional to PfATP4 S374R confer enhanced resistance to MB14 we performed parasite proliferation assays with the clonal lines A5, A6 and D14 that had additional mutations, and with the lines B7, B8, D11, E15 and E16 that had none. The 72 h growth assays were performed in triplicate with the SYBR green nucleic acid stain used for the quantification of surviving parasites, since this method was better suited to larger sample sizes. The mean EC_50_ for growth inhibition by MB14 was not significantly different (p > 0.05, one way ANOVA) between the parasite lines with the single S374R PfATP4 mutation and those lines with the additional mutations, suggesting that mutations additional to S374R did not appear to be substantially enhancing MB14 resistance (Fig. 2). We note that the EC_50_ values for inhibition of parasite growth by MB14 estimated using the SYBR green method were higher than those estimated using the LDH method, and that the differences between the 3D7 and MB14r clones were not as great as those seen using the LDH method. Although the MB14 EC_50_ of PfATP4 S374R parasites was always higher than that for the clonal 3D7 lines 3D7-1 and 3D7-2, the difference was not always statistically significant for some of the lines tested (Fig. 2). When combined, the mean EC_50_ for all the S374R mutants (23.1 ± 1.5 µM; mean ± SD, n = 24) was significantly higher (p < 0.0001) than that for the 3D7 clones (6.19 ± 0.48 µM; mean ± SD, n = 6).

### MB14 resistant parasites are cross-resistant to a related compound and another PfATP4 inhibitor

To determine whether the PfATP4 S374R mutation also conferred reduced parasite susceptibility to the related compound MB10 (also called compound 23^24^) we performed 72 h growth assays with this compound (Fig. 2). The MB10 EC_50_ values for the MB14r parasite clones were, in each case, higher than those for the 3D7 clones. Although differences were not significant for comparisons of some MB14r clones with 3D7 clones, the combined mean EC_50_ of 8.07 ± 0.54 µM (mean ± SD, n = 24) for MB14r parasites was significantly higher (p < 0.0001) than the EC_50_ for the 3D7 parasites (1.81 ± 0.54; mean ± SD, n = 6; Fig. 2).

We also assessed the sensitivity of the MB14r parasite lines to another growth-inhibitory compound, RK18, which is similar to MB14 and MB10 but contains an aniline substituent at R7 of isoquinoline. RK18 inhibits the growth of 3D7 parasites with similar potency to MB14 and MB10 (Kalhor and Abbott, manuscript in preparation). The combined mean EC_50_ for inhibition of the growth of the MB14r parasite clones by RK18 was 3.55 ± 0.57 µM (mean ± SD, n = 24), not significantly different from that for inhibition of the growth of the parental 3D7 clones (2.64 ± 0.40 µM; mean ± SD, n = 6; p = 0.441). This is consistent with RK18 inhibiting parasite growth via a different mechanism of action than MB14 or MB10 (Fig. 2).

Parasites with resistance-conferring mutations in PfATP4 commonly show cross-resistance to a structurally-diverse range of PfATP4-associated compounds^33–35,37^. To investigate whether the MB14r parasite lines isolated here show such cross-resistance we investigated their sensitivity to the PfATP4 inhibitor cipargamin which is the most well-developed of the spiroindolone class of antimalarials^28^ and which is currently undergoing clinical trials^43,44^. Proliferation of the wildtype 3D7 parasites was inhibited by cipargamin with an EC_50_ of 1.12 ± 0.11 nM (mean ± SD, n = 6), similar to previous reports (Fig. 2)^28^. The MB14 resistant parasites showed a significantly reduced sensitivity (p < 0.0001) with a combined mean EC_50_ of 2.90 ± 0.12 nM (mean ± SD, n = 24).

### MB14 inhibits PfATP4-associated ATPase activity

**Figure 3:**
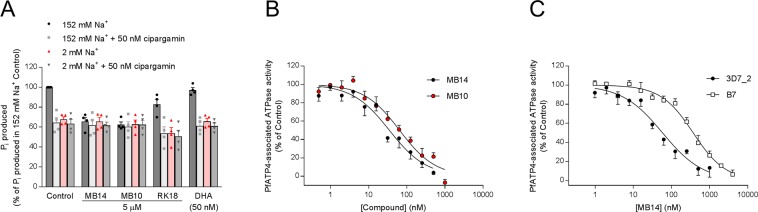
MB14 and its analog MB10 inhibited Na^+^ dependent ATPase activity in *P. falciparum* membrane preparations consistent with the compounds targeting PfATP4. (**A**) The effects of various compounds on *P. falciparum* membrane ATPase activity under high-[Na^+^] (152 mM) and low-[Na^+^] (2 mM) conditions in the presence and absence of cipargamin (50 nM). Dihydroartemisinin (DHA) was tested at a concentration of 50 nM; MB14, MB10 and RK18 were each tested at a concentration of 5 μM. The data were obtained with 3D7 parasites and the bars show the mean (+SEM) from four independent experiments, each performed on different days with different membrane preparations. The symbols show the data from the individual independent experiments. The P_i_ produced is shown as a percentage of that measured in the 152 mM Na^+^ Control. P_i_ production in the 152 mM Na^+^ Control varied from 30 to 86 nmol per mg (total) protein per min in the different experiments. For each compound, the (pre-normalised data) were tested for statistical significance compared to the Control (0.4% v/v DMSO or 50 nM cipargamin only) data for the same (high-[Na^+^] or low-[Na^+^]) condition; *p < 0.05, **p < 0.01, ***p < 0.001 (one-way ANOVA with ‘experiment’ nominated as a ‘blocking factor’). (**B**) Potency of MB14 (black circles) and MB10 (red circles) against PfATP4-associated ATPase activity in membranes from 3D7 parasites. The data are the mean (shown + or − SEM) obtained from five independent experiments (with the exception of the highest and the lowest concentrations of MB10, for which data are n = 2 and n = 3, respectively). (**C**) Potency of MB14 against PfATP4-associated ATPase activity in membranes from B7 parasites expressing PfATP4^S374R^ (white squares) and matched control parasites expressing wild-type PfATP4 (3D7_2; black circles). The data are the mean (shown + or − SEM) obtained from 5–6 independent experiments (with the exception of the two highest concentrations of MB14 in B7 parasites, for which data are n = 2). In (**B** and **C**), the PfATP4-associated ATPase activity was calculated by subtracting the total ATPase activity measured in the presence of 50 nM cipargamin from that measured in the absence of cipargamin. Each experiment was performed on a different day with a different membrane preparation.

PfATP4 is a plasma membrane P-type ATPase that has been proposed to function as a Na^+^ efflux pump, extruding Na^+^ ions from the parasite cytosol while importing H^+^ ions^26^. To investigate whether MB14 and its analogues target PfATP4, we examined their effects on the cipargamin-sensitive Na^+^-ATPase activity present in *P. falciparum* membrane preparations. Each compound was tested for its effect on the rate of ATP hydrolysis, estimated from the rate of production of inorganic phosphate (P_i_), by membranes prepared from isolated parasites. Each compound was tested under high-[Na^+^] (152 mM) and low-[Na^+^] (2 mM) conditions and in the presence and absence of cipargamin at a concentration sufficient to cause full inhibition of the Na^+^-dependent ATPase activity (50 nM). MB14 and MB10 both inhibited ATPase activity in the high-[Na^+^] condition when cipargamin was not present (Fig. 3A,B), but were without effect on ATPase activity under low-[Na^+^] conditions or in the presence of cipargamin. Both compounds therefore inhibited the Na^+^-dependent cipargamin-sensitive ATPase (equated with PfATP4) present in parasite membranes. By contrast, RK18 caused a decrease in ATPase activity under each of the conditions tested (Fig. 3A), but had no net effect on the amount of P_i_ produced by Na^+^-dependent ATPase activity (measured as 2.19 ± 0.43 nmol in the presence of RK18 and 2.42 ± 0.37 nmol for the control; mean ± SEM, n = 4; *p* = 0.3; paired t-test). This is consistent with RK18 not inhibiting PfATP4 but inhibiting a Na^+^-independent ATPase associated with the parasite membranes. Dihydroartemisinin (50 nM; ~16 × IC_50_ for growth inhibition of 3D7 parasites^45^) was without effect on membrane ATPase activity under any of the conditions tested (Fig. 3A).

We next investigated the concentration-dependence of the inhibition of the cipargamin-sensitive fraction of parasite membrane ATPase activity (the ‘PfATP4-associated ATPase activity’) by MB14 and MB10^38^. Each concentration of MB14 and MB10 was tested in the presence and absence of 50 nM cipargamin, and the amount of P_i_ produced in the presence of cipargamin was subtracted from that produced in its absence to yield the cipargamin-sensitive (PfATP4-associated) ATPase activity. Both MB14 and MB10 inhibited PfATP4-associated ATPase activity in membranes from 3D7 parasites, with IC_50_ values of 52 ± 16 nM (mean ± SEM, n = 5) and 62 ± 9 nM (mean ± SEM, n = 5), respectively (Fig. 3B).

We then compared the potency of MB14 against the membrane ATPase activity associated with PfATP4^S374R^ (using membranes prepared from B7 parasites) and wild-type PfATP4 (using membranes prepared from 3D7_2 parasites). In membranes prepared from 3D7_2 parasites, MB14 inhibited PfATP4-associated ATPase activity with an IC_50_ value of 70 ± 19 nM (mean ± SEM, n = 6). MB14 was 6-fold less potent at inhibiting PfATP4-associated ATPase activity in membranes from B7 parasites (Fig. 3C), doing so with an IC_50_ value of 429 ± 73 nM (mean ± SEM, n = 5; *p* < 0.001; unpaired t-test). This provides further evidence that MB14 targets PfATP4, and that mutations in PfATP4 confer resistance to MB14 by reducing the ability of the compound to bind to or otherwise inhibit the transporter.

### Inhibition of PfATP4 induces partial lysis of infected erythrocytes

**Figure 4:**
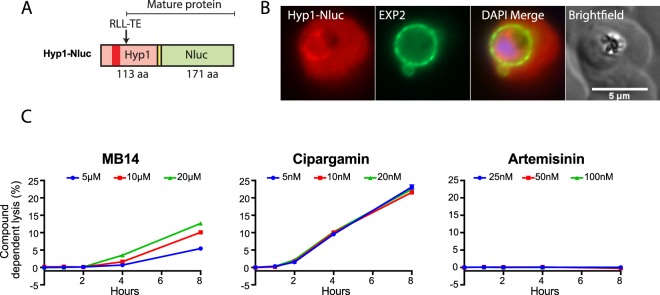
PfATP4 inhibitors induce the lysis of erythocytes infected with nanoluciferase reporter parasites. (**A**) Diagram of the nanoluciferase reporter N-terminally appended to the export signal region from the parasite PEXEL protein Hyp1 (PF3D7_0113300) that was used to transfect wildtype 3D7 reporter parasites. The RLL-TE PEXEL cleavage site is shown to indicate the mature form of the reporter protein. (**B**) Hyp1-Nluc expressing trophozoite stage infected erythrocyte following 0.04% v/v DMSO treatment showing export of the reporter protein into the host erythrocyte compartment. The cells were probed with rabbit anti-nanoluciferase and a mouse monoclonal antibody for parasitophorous vacuole marker protein EXP2. DNA stained with DAPI (4,6-diamidino-2-phenylindole). (**C**) Lysis of parasitized erythrocytes was ascertained following a treatment time course with MB14 (5, 10, 20 µM), cipargamin (5, 10, 20 nM) and control compound artemisinin (25, 50, 100 nM). Compound dependent cell lysis (%) was estimated by measuring the amount of Hyp1-Nluc released into the media relative to that in the whole culture minus the DMSO vehicle (~1% at 8 h). The data are from a single experiment, and are representative of those obtained in three independent experiments, each performed in triplicate.

Cipargamin and other PfATP4-associated compounds induce swelling of isolated intracellular parasites and parasitised erythrocytes, and some lysis of trophozoite- and schizont-infected erythrocytes^29,35,36^. To determine whether MB14 also induces lysis of infected cells, parasites were transfected with a reporter construct in which the Hyp1 PEXEL protein was fused to nanoluciferase (Hyp1-Nluc), resulting in the fusion protein being exported into the erythrocyte compartment^46^ (Fig. 4A,B). To estimate lysis over an 8 h drug treatment period, the media which contained Hyp1-Nluc trophozoite infected erythrocytes was replaced with fresh media containing the various compounds (at concentrations of about 5×, 10× and 20 × EC_50_ for inhibition of parasite growth using the LDH growth assay). The media was harvested 0, 1, 2, 4 and 8 h later and the degree of cell lysis was ascertained by measuring the levels (in relative light units; RLU) of Hyp1-Nluc released into the media by lysis or leakage. This was then divided by the RLU of total culture (cells and media, with the cells fully lysed in NanoGlo assay reagent) for each time point to estimate percentage cell lysis.

In infected cells exposed to MB14 there was, at all concentrations tested (up to 20 µM), no detectable lysis for the first 2 h. From 2 h MB14-treated cells underwent progressive lysis in a time- and concentration-dependent manner (Fig. 4C). For each drug concentration, the background lysis of the Hyp1-Nluc infected erythrocytes treated with the DMSO vehicle (~1% lysis at 8 h) was subtracted in order to obtain the degree of MB14-dependent lysis (Fig. 4C). At the highest MB14 concentration tested (20 µM) 10–15% of the infected cells had undergone lysis after 8 h. For infected cells treated with cipargamin (5, 10 and 20 nM) there was again an initial lag of approximately 2 h, after which there was progressive lysis, with the infected cells undergoing a greater degree of lysis than was seen with MB14; 20–25% of the infected cells had undergone lysis after 8 h of treatment at all three concentrations (Fig. 4C). The potent, rapidly-acting antimalarial artemisinin was also assayed at three concentrations (25, 50 and 100 nM) and was found to cause no appreciable lysis of Hyp1-Nluc trophozoite infected erythrocytes. The data are consistent with the induced leakiness or lysis of infected erythrocytes being specific to PfATP4 inhibitors.

MB14, when used at 20 µM, did not appear to lyse uninfected erythrocytes since the culture media did not turn pink with hemoglobin after 8 h. We also examined the toxicity of MB14 on HepG2 and HEK293 cell lines and although the viability of these cell lines declined at 50 and 100 µM MB14, at 25 µM the cells grew normally suggesting that the 20 µM concentration used in the *P. falciparum* lysis assay was not generally toxic to the host cells (Fig. S8).

### The MB14-induced lysis of parasitised erythrocytes is dependent on the new permeability pathways in the infected erythrocyte membrane

The intraerythrocytic malaria parasite induces in the erythrocyte membrane ‘new permeability pathways’ (NPPs) that confer upon the membrane an increased permeability to a wide range of low molecular weight solutes: nutrients, metabolic wastes, and (monovalent) inorganic ions, including Na^+^ ^47^. The NPPs serve as the primary route of entry of Na^+^ into the parasitised erythrocyte^48^. NPP formation has recently been reported to depend on the RhopH complex of parasite proteins that are introduced into the erythrocyte during invasion and that later relocate to the erythrocyte membrane^49–51^. To investigate the role of the NPPs in the leakage/lysis of infected cells that is induced by inhibition of PfATP4 we knocked down expression of RhopH2 (one of the three proteins of the RhopH complex) and measured the susceptibility of parasites to PfATP4 inhibitor-induced lysis.

**Figure 5:**
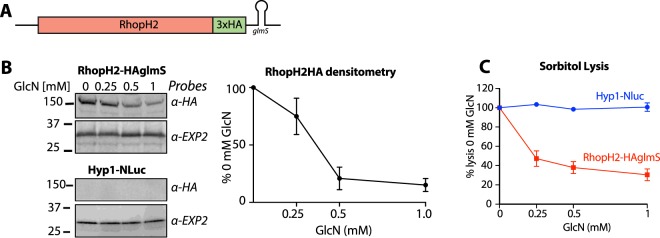
Reduction of RhopH2 expression reduces the lysis of infected erythrocytes by sorbitol entry via new permeability pathways (NPPs). (**A**) Diagram of the *rhopH2* locus tagged with *haglmS*. (**B**) Trophozoite stage Hyp1-Nluc and RhopH2-HAglmS infected erythrocytes (IE) were treated with the concentrations of glucosamine (GlcN) indicated for 48 h days to reduce the expression of RhopH2-HA in RhopH2-HAglmS parasites. Densitometry of western blot bands from three independent experiments are shown with each point indicating mean and SD. Full size blots can be viewed in Fig. S9. (**C**) The reduction of RhopH2-HA expression reduced the susceptibility of RhopH2-HAglmS infected cells to sorbitol induced lysis relative to Hyp1-Nluc 3D7 infected control cells. The lysis of IE was quantified by measuring the bioluminescence of Hyp1-Nluc released from the infected IE.

To knockdown RhopH2, the gene was appended to the sequence of a self-cleaving *glmS* ribozyme that reduces mRNA and hence protein levels (Fig. 5A)^49^. The protein was also labelled with a hemagglutinin (HA) epitope tag for detection with anti-HA IgG. Upon addition of glucosamine (GlcN) there was a concentration dependent reduction in RhopH2-HA expression (Figs 5B and S9). Western blot densitometry of RhopH2-HAglmS parasites treated with 1 mM GlcN for one cell cycle, from trophozoites to trophozoites, revealed an approximately 80% reduction of RhopH2-HA expression (Fig. 5B). The EXP2 loading control indicated that nearly identical amounts of parasite material were present in each lane and that parasite growth was not substantially slowed by the 48 h GlcN treatment (Figs 5B and S10). The RhopH2-HAglmS parasites were also transfected with the Hyp1-Nluc reporter to quantify cell lysis and are here referred to as RhopH2-HAglmS parasites to distinguish them from Hyp1-Nluc parasites made in the wildtype 3D7 background described above.

Lysis of infected erythrocytes in isotonic 280 mM sorbitol solution is dependent upon the formation of NPPs (sorbitol enters the infected erythrocytes via these pathways, inducing osmotic lysis) and may be monitored using a luminometer to measure the release of Hyp1-Nluc reporter protein into sorbitol media containing its NanoGlo substrate. RhopH2-HAglmS trophozoite-stage parasitised erythrocytes were resuspended in sorbitol media and the RLU was recorded every 3 min for up to 1 h. As lysis of infected erythrocytes proceeded and released more nanoluciferase into the media, the RLU increased, allowing the determination of the rate of lysis (RLU/min). The RLU/min of erythrocytes not treated with GlcN was normalised to 100%^49,52^, reflecting the normal level of lysis in cells infected with RhopH2-HA expressing trophozoites. Zero percent lysis was defined as RLU/min measured for infected erythrocytes in non-lytic phosphate buffered saline. Sorbitol lysis declined substantially in cells in which the levels of RhopH2-HA were reduced following GlcN treatment (Fig. 5B,C). By contrast, the lysis of cells infected with Hyp1-Nluc control parasites was unaffected by GlcN, reflecting the fact that these cells maintained normal levels of RhopH2 (though this could not be confirmed due to lack of a protein specific antibody (Fig. 5B))^49^. Together, these data demonstrate that NPP activity can be reduced through the inducible knockdown of RhopH2.

**Figure 6:**
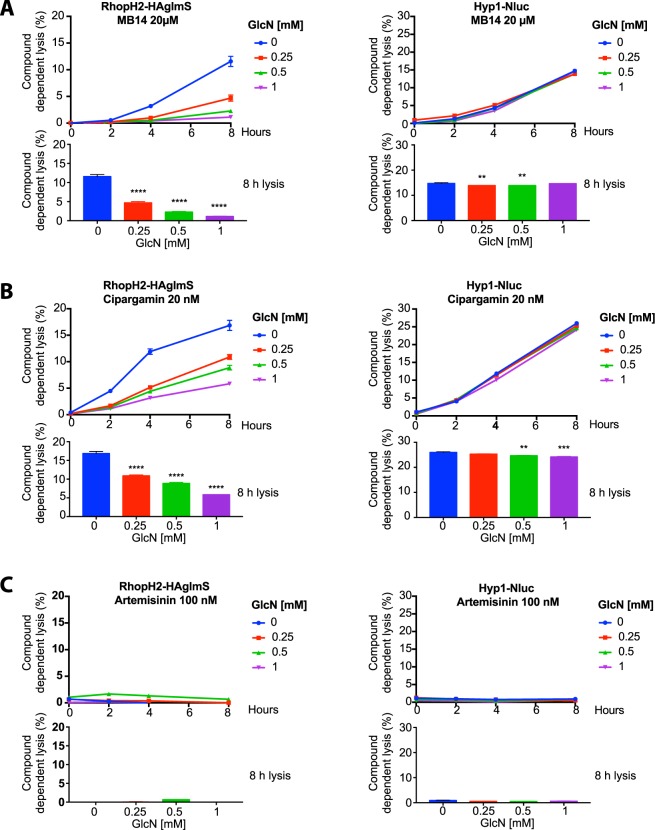
Knockdown of RhopH2-HA reduces lysis of erythrocytes infected with RhopH2-HAglmS trophozoites. RhopH2-HAglmS and control Hyp1-Nluc trophozoite-infected erythrocytes were treated for 48 h with the concentrations of GlcN indicated to knockdown expression of RhopH2-HA in the RhopH2-HAglmS cells. The trophozoite-infected erythrocytes were then washed and treated with (**A**) MB14 (20 µM), (**B**) cipargamin (20 nM) and (**C**) artemisinin (100 nM) and incubated from 0 to 8 h in their respective compounds. After sampling at 0, 2, 4 and 8 h the % Compound dependent lysis was obtained by dividing the RLU produced by nanoluciferase in the media by the RLU of the whole culture (media and cells). Background levels of lysis measured in the 0.04% v/v DMSO drug vehicle control wells (~1%) were subtracted from the compound induced values to measure specific compound-induced lysis. One representative set of graphs from three biological repeats is shown; each experiment contained three technical repeats. The data show the mean and SD. The top graph for each drug treatment shows lysis versus time post drug treatment and the bottom bar graphs show lysis at 8 h with asterisks indicating significant differences between GlcN and untreated parasites. One way ANOVA with Dunnett’s multiple comparisons test; **p < 0.01, ***p < 0.001, ****p < 0.0001.

To determine if inhibition of NPP formation by RhopH2-HA knockdown reduced the lysis induced by PfATP4 inhibitors, the GlcN-treated parasitised erythrocytes were incubated for up to 8 h in 20 × EC_50_ (for growth) of each of the different PfATP4 inhibitors of interest. For RhopH2-HAglmS and Hyp1-Nluc trophozoite-infected erythrocytes not treated with GlcN, 20 µM MB14 induced time-dependent lysis (Fig. 6A). After 8 h of exposure to MB14 approximately 12% of the RhopH2-HAglmS infected erythrocytes had undergone lysis, similar to the 15% lysis observed in Hyp1-Nluc infected erythrocytes (Fig. 6A). In contrast, lysis of RhopH2-HAglmS infected erythrocytes treated with GlcN for one cell cycle to knockdown RhopH2-HA was reduced after a 2 h exposure to 20 µM MB14 (Fig. 6A). Knockdown of RhopH2-HA with 0.25, 0.5 and 1 mM GlcN reduced MB14-induced lysis after 8 h from 12% to about 4%, 2% and 1% respectively (p < 0.0001, one of 3 biological replicates is shown in Fig. 6A). By contrast, GlcN-treatment of erythrocytes infected with Hyp1-Nluc control parasites caused just a slight reduction in lysis which, although significant (p < 0.01), was much lower than the reduction observed in erythrocytes infected with RhopH2-HAglmS parasites following GlcN treatment (Fig. 6A).

Treatment of parasitised erythrocytes with 20 nM cipargamin produced a similar trend to that seen with MB14 (Fig. 6B). After an 8 h cipargamin exposure approximately 17% of RhopH2-HAglmS-infected cells not treated with GlcN had undergone lysis. This decreased significantly (p < 0.0001) to 5% lysis in RhopH2-HAglmS-infected cells treated with 1 mM GlcN (Fig. 6B). GlcN treatment at 0.25 and 0.5 mM produced an intermediate reduction of lysis. Treatment with the control drug artemisinin resulted in minimal lysis of the infected erythrocytes, barely above the background lysis of the DMSO vehicle control (Fig. 6C).

To confirm that the bioluminescence activity measured after PfATP4 inhibitor treatment was due to the lysis of infected erythrocytes, thin blood smears of the cells were examined after 8 h of exposure to each of the different compounds. MB14 and cipargamin treatment, but not artemisinin treatment, caused the centres of parasites to stain less well than the cell periphery, suggesting that the parasites had become vacuolated or ‘swollen’ (Fig. S11A). The parasitemias of RhopH2-HAglmS samples treated with (lysis inducing) MB14 and cipargamin were lower than those treated with artemisinin, which we have shown does not cause lysis (Fig. 6). The MB14- and cipargamin-induced reduction in RhopH2-HAglmS parasitemia was not observed following 1 mM GlcN treatment (Fig. S11B). For Hyp1-Nluc parasites, MB14 and cipargamin reduced parasitemia relative to artemisinin however this reduction was observed regardless of whether or not cells were exposed to GlcN (Fig. S11B). The morphological data therefore corroborate the bioluminescence data.

**Figure 7:**
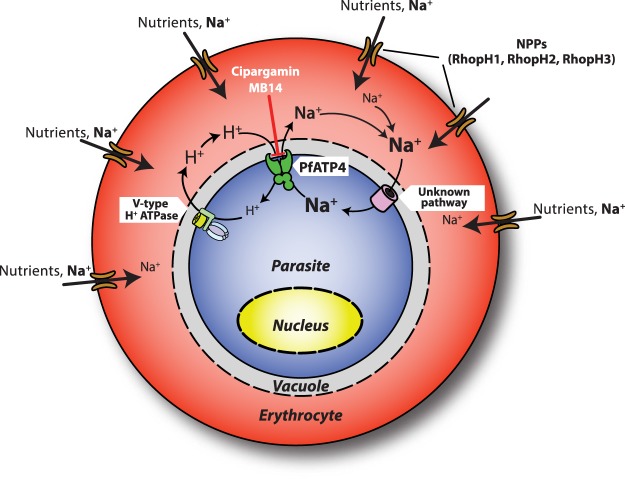
Proposed role of the parasite-induced New Permeability Pathways (NPPs) in the lysis of parasitized erythrocytes following inhibition of PfATP4 by MB14 or cipargamin. Inducible knockdown of RhopH2-HAglmS reduces NPP activity and the infected erythrocytes thereby become resistant to sorbitol lysis. The NPPs serve as the major route of entry of Na^+^ into the parasitized erythrocyte^48^. Reduced expression of the NPP results in reduced entry of Na^+^ into the infected erythrocyte, reduced uptake of Na^+^ by the parasite following PfATP4 inhibition, reduced osmotic swelling of the parasite and hence reduced swelling and lysis of the infected cell.

Together, the data are consistent with NPPs playing a role in the MB14- and cipargamin-induced lysis of infected cells. A mechanism for this is that the Na^+^ which enters the intra-erythrocytic parasite, and causes it to swell following inhibition of PfATP4, gains access to the infected erythrocyte via the NPPs. Reduced expression of the NPPs results in reduced Na^+^ influx into the infected cell, reduced uptake of Na^+^ by the parasite, reduced parasite swelling and hence reduced lysis of the infected cell. This is illustrated schematically in Fig. 7.

An alternative possibility is that MB14 and cipargamin gain entry to the infected erythrocyte via NPPs, and that RhopH2 knockdown reduces the access of the inhibitors to the intra-erythrocytic parasite. The latter possibility was investigated using furosemide, an effective chemical inhibitor of the NPPs. Cells infected with RhopH2-HA parasites that had not been pre-treated with GlcN (and which therefore expressed the NPPs at the normal level) were treated with either 20 µM MB14 or 20 nM cipargamin for 20 min, allowing the compounds time to enter the infected erythrocytes and reach their target. The cells were then suspended in either the presence or absence of 100 µM furosemide, to inhibit the NPP-mediated influx of Na^+^ into the infected erythrocytes^48^. Cell lysis was monitored via nanoluciferase activity as above after 2, 4 and 8 h.

**Figure 8:**
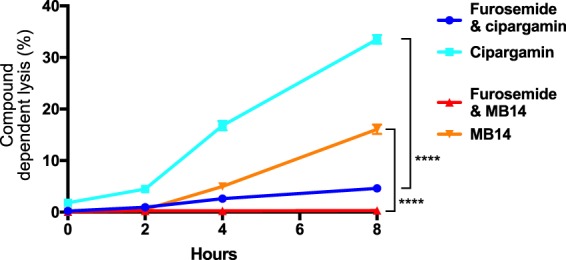
The NPP-blocking compound furosemide protects parasite infected erythrocytes from lysis induced by PfATP4 inhibitors cipargamin and MB14. Erythrocytes infected with RhopH2-HAglmS trophozoites expressing exported nanoluciferase were treated with either 20 µM MB14 or 20 nM cipargamin for 20 min then resuspended in medium in either the presence or absence of 100 µM furosemide. Compound dependent lysis is the percentage of relative light units (RLU) produced by nanoluciferase released into the media from lysed infected RBCs divided by the total RLU of the whole culture (media and cells). One representative experiment of the three performed (each of which contained three technical repeats) is shown here. Error bars indicate SD, ****p < 0.0001, unpaired T-test.

As can be seen in Fig. 8, furosemide caused a significant suppression of both the MB14-induced and the cipargamin-induced lysis of parasitised erythrocytes. This is consistent with the NPPs playing a key role in the phenomenon, most likely in mediating the influx of Na^+^ into the infected cells. Inhibition of the NPPs by furosemide slows the influx of Na^+^, thereby slowing the Na^+^-induced swelling of the intraerythrocytic parasite and the consequent swelling (and, ultimately, lysis) of the infected cell.

## Discussion

To find the biological target of the 4-cyano-3-methylisoquinoline MB14 compound in *P. falciparum* we selected for genetic resistance and identified a S374R mutation in the parasite’s plasma membrane Na^+^ efflux pump PfATP4. This pump serves to maintain a low cytosolic [Na^+^] in the parasite, countering the influx of Na^+^ into the parasite, down its electrochemical gradient, via unknown pathways^26^. On the basis of homology modelling of PfATP4, based on the known structure of the related human Ca^2+^ ion pump, SERCA, the resistance-conferring S374R mutation identified here is predicted to be on the end of transmembrane helix 3. Many SERCA inhibitors such as thapsigargin are thought to bind to the transporter’s transmembrane helices in the E2 state, preventing them from undergoing conformational change to return to the E1 state^53^; this may be how MB14 functions. The S374R mutation could reduce the affinity with which MB14 binds to the protein in the vicinity of transmembrane helix 3 and/or reduce the access of the compound to other helices within the transporter.

One feature of some PfATP4 mutant parasites is that they grow more slowly^35^, possibly as a result of higher resting [Na^+^] in the parasite cytosol^26,35^. Previously, selection for resistance to the pyrazoleamide PA21A050 resulted in mutations in PfATP4 (V178I) and Ca^2+^-dependent protein kinase PfCDPK5 (T392A), which together conferred greater resistance than did either mutation alone^36^. In the same study it was observed that trophozoite-stage parasites within PA21A050-treated infected erythrocytes appeared to swell and in some cases burst, possibly due to the accumulation of Na^+^ in the parasite cytosol and the osmotic uptake of water. PfCDPK5 regulates schizont rupture and the egress of merozoites^54^ and so mutations in the kinase may reverse premature rupture triggered by accelerated swelling of schizont infected erythrocytes^36^. Parasite swelling has been observed in parasites treated with numerous PfATP4 inhibitors including structurally diverse compounds identified in the MMV Malaria and Pathogen boxes^29,34^. At 10 µM MB14 triggers premature schizont rupture and consequently reduced invasion^24^ and it is possible that the mutations additional to PfATP4 S374R serve to reduce premature lysis. Comparison of the potency of MB14 against MB14 resistant S374R mutants that only contained the single PfATP4 mutation (clonal lines B7, B8, D11 and E16) and clonal lines that possessed additional mutations (A5, A6 and D14) indicated that although there were differences in mean EC_50_ between the lines these were not statistically significant. These data are consistent with the mutations additional to PfATP4 S374R being random in nature, or if selected for, not conferring significant additional resistance to MB14.

Both MB14 and the structurally similar compound MB10 inhibited PfATP4-associated ATPase activity, with EC_50_ values of 50–70 nM in membranes from parasites expressing wild-type PfATP4. The EC_50_ value for cipargamin against PfATP4-associated ATPase activity (12 nM in membranes from wild-type Dd2 parasites^38^) is only 4–5 fold lower than that of MB14 and MB10, yet cipargamin is about 1000 times more potent than MB14 and MB10 at inhibiting parasite growth^38^. Cipargamin induces faster and greater parasite lysis than MB14 at 1000 times lower concentration (Fig. 6). MB14 and MB10 are more potent against PfATP4-associated ATPase activity than against parasite growth, whereas the opposite is true for cipargamin. There are a variety of possible explanations for a difference in the potency of a compound against its target (as measured in a cell-free assay) and against whole cells. The concentrations of MB14 and MB10 that are attained in the cytosol of intraerythrocytic parasites are not known, and may be lower than those in the extracellular medium. The reverse may be true for cipargamin (i.e. it might accumulate in parasites and reach concentrations higher than those in the culture medium).

There are numerous reports of PfATP4-resistance mutations selected by one PfATP4-associated compound conferring cross-resistance to other PfATP4 inhibitors^33–35,37^. Our MB14 resistant PfATP4 S374R parasites were about 2.5-fold less susceptible to cipargamin than the parental 3D7 parasite line. The basis for this cross-resistance is not known but it is likely that the mutation alters the structure of PfATP4 in such a way as to reduce the binding efficacy of cipargamin.

Inhibition of PfATP4 leads to parasite swelling^29,34,36^ and we have shown here that infected erythrocytes became leaky and/or lysed after treatment with MB14 and cipargamin. Artemisinin caused much less lysis than the PfATP4-associated compounds, even though it causes cellular damage^55^. Treatment with another PfATP4 inhibitor (+)-SJ733 causes both parasite and host cell lysis and this in combination with an increase in cytosolic [Na^+^] and cytosolic alkalinisation probably contributes to parasite killing^35^. Although parasite lysis may be one of the mechanisms of parasite death in *in vitro* culture, in an *in vivo* setting, cipargamin clears parasites rapidly with half-life clearance times of less than an hour^44^. Cipargamin induces swelling of both parasites and parasitised erythrocytes within minutes of its addition, raising the possibility that splenic clearance of rigid and swollen infected erythrocytes may be behind the mechanism of rapid parasite clearance *in vivo*^29^.

Although it has long been known that Na^+^ gains entry into infected erythrocytes via the NPPs and eventually overwhelms the capacity of the erythrocytes to pump the ion out^48^, proteins that comprise the NPPs have only recently been identified. RhopH1, and later RhopH2 and RhopH3 have been implicated in NPP function, with the former likely to form the channel that spans the erythrocyte plasma membrane^49–51,56,57^. We have shown that lysis of infected erythrocytes induced by PfATP4 inhibitors was reduced in parasites in which RhopH2 was knocked down or in which the NPPs were blocked with furosemide. This is consistent with the NPPs playing a key role in mediating the influx into the infected cell of the Na^+^ ions that accumulate in parasites treated with PfATP4 inhibitors. It is interesting that mutations in RhopH proteins that would limit Na^+^ entry into the erythrocyte have not been recovered following selection for resistance to PfATP4 inhibitors. Mutations conferring resistance to compounds that block NPP function led to the first evidence that parasite proteins were specifically required for NPP activity^56^. Perhaps mutations that reduce Na^+^ entry into the erythrocyte also reduce the uptake of key nutrients, thereby restricting parasite growth and preventing the recovery and identification of such mutations.

## Methods

### Materials

MB14 and MB10 were synthesised by R. Kalhor, M. Buskes and B. Abbott as described previously^24^. The same authors provided RK18 and the method of its synthesis is currently being prepared for publication. Cipargamin was kindly provided by the Medicines for Malaria Venture. Furosemide, glucosamine, artemisinin and dihydroartemisinin were from Sigma.

### Parasite culture

*Plasmodium falciparum* strain 3D7 was cultured in human RBCs (Australian Red Cross Blood Bank, blood-group O^+^) at 4% haematocrit in complete RPMI media (RPMI-HEPES, 0.5% AlbuMaxII [GIBCO], 0.2% NaHCO_3,_ 0.37 mM hypoxanthine) at 37 °C as described previously^58^. A plasmid containing Hyp1-Nluc under an *eflα* promoter was transfected into erythrocytes and these were inoculated with erythrocytes infected with 3D7 and RhopH2-HAglmS trophozoites^49^ as described previously^59^. Transfected parasites were selected with blasticidin S (Sigma) at 5 µg/mL.

### Resistance selection

After a clonal population of 3D7 parasites was isolated by limiting dilution the parasites were cultured to 5% parasitemia in 4% haematocrit suspensions as indicated above and 5 mL of culture (~1 × 10^8^ parasites) was added to each well of a six well tissue culture plate. Five of the six wells were treated with 10 µM MB14 (~10 × EC_50_) in DMSO and the sixth control well was treated with 0.1% v/v DMSO (solvent control). Every two days the culture media and MB14 or DMSO was replaced and the culture monitored by microscopy. Once most of the parasites had appeared to die the MB14 was removed and the cultures were maintained in MB14-free culture media. Once the population had recovered MB14 treatment was resumed. A total of six treatment cycles were performed with the time to parasite death becoming longer and the recovery period becoming shorter. After cycles 3 and 6 the EC_50_ of MB14 was assessed by a 72 h growth assay using lactate dehydrogenase activity to monitor parasite biomass^30^.

### Library preparation and genome sequencing

In the absence of drug treatment, the MB14 resistant parasites and the parental 3D7 parasites were cloned by limiting dilution. After assaying for the maintenance of drug resistance, genomic DNA was prepared from MB14 resistant (and parental) saponin-lysed parasites using a DNeasy Blood and Tissue Kit (Qiagen). An input of 200 ng of genomic DNA was prepared and indexed for Illumina sequencing using the TruSeq DNA sample Prep Kit (Illumina) as per the manufacturer’s instructions. The library was quantified using the Agilent Tapestation and the Qubit™ RNA assay kit for Qubit 2.0® Fluorometer (Life technologies). Sequencing was performed on an Illumina NextSeq 500 instrument using the v2 150 cycle High Output kit (Illumina) as per manufacturer’s instructions. All samples were sequenced in the same run. Indexed libraries were sequenced using 2 × 81 cycles paired-end plus a 6 base index read. Sequences were aligned to reference PlasmoDB-29_Pfalciparum3D7, using bowtie2 version 2.2.9^60^, with parameter –-sensitive-local. Duplicate reads were removed using Picard tools MarkDuplicates version 2.9.4^61^.

Base calling and quality scoring were performed with Real-Time Analysis v2.4.6. FASTQ file generation and de-multiplexing was performed with bcl2fastq conversion software v2.15.0.4. Copy number analysis was done with the R package QDNAseq, version 1.14.0^62^, with bin sizes of 5 kbp and 10 kbp. All samples showed same coverage pattern as the control lines, so that relative copy numbers, normalised to the 3D7 control lines, had no deleted or highly amplified regions (Fig. S12). Structural variants were called using GRIDSS v1.4^31^. The only rearrangements were in telomeric regions or in regions local to genes for erythrocyte membrane proteins. Two different small event callers (SNVer v0.5.3^63^ and VarScan2 v2.4^64^, with –-min-coverage 10 –-min-reads), were used to find single nucleotide variants (SNVs) and small insertions and deletions that were not in the parental 3D7 clones. Initial calls were filtered to require depth in all samples at that location to be at least 10 and alternate allele frequency in at least one sample to be greater than 0.5, to remove spurious calls. A single non-synonymous SNV was consistently found in the protein coding sequence of *pfatp4* (S374R) in all clones of the four resistant lines, but not in 3D7 clones. Another three SNVs were identified in certain MB14 resistant clones as described in the Results.

The presence or absence of the four SNVs identified in the parasite lines was confirmed by amplifying a ~0.5 kb regions covering the mutations from genomic DNA. All four mutations were validated in two wildtype 3D7-1 and 3D7-2 clones and MB14 resistant clones A5, A6, B7, B8, D11, D12, E15 and E16. PCR products were directly sequenced from gel-excised bands and the primers used are listed in Table S2.

### Growth inhibition assays

To measure growth inhibition of our parasite lines by the various compounds of interest, media containing the compounds over a range of concentrations was added to suspensions of parasitised erythrocytes (0.3% parasitemia comprising predominately ring-stage parasites; 2% haematocrit) with the final DMSO concentration not exceeding 0.2% v/v. One hundred µL aliquots of parasite cultures were grown in 96 well plates for ~72 h followed by measurement of lactate dehydrogenase activity as a marker of parasite proliferation^30^. The OD650 nm values of control uninfected blood samples were subtracted from the infected erythrocyte values and the EC_50_ values were derived from plotting drug concentrations against OD650 values in GraphPad Prism.

Where large numbers growth inhibition assays needed to be performed 50 µL of 72 h compound-treated parasite culture was mixed with 50 µL of SYBR green lysis buffer, (5 mM EDTA, 0.008% saponin, 0.08% Triton X-100 and 20 mM Tris pH 7.5) containing 0.2 µL SYBR green (Molecular Probes) per mL lysis buffer in black 96 well plates. After 1 h incubation, fluorescence was measured in a Clariostar plate reader (BMG Labtech) using an excitation wavelength of 485 nm and an emission wavelength of 528 nm.

### Measurements of membrane ATPase activity

Membranes were prepared from saponin-isolated mature trophozoite-stage parasites as described previously^38^. ATPase activity was determined by measuring the production of P_i_ from ATP hydrolysis, using the PiColorLock Gold Phosphate Detection System (Innova Biosciences). The duration of the reactions was 10 min and the reaction mixtures had a pH of 7.2 and contained 20 mM KCl, 2 mM MgCl_2_, 50 mM Tris, 150 mM NaCl or choline chloride, parasite membrane (final concentration of total protein was 50 μg/mL), the compound(s) of interest (final [DMSO] in each reaction = 0.5% v/v) and 1 mM ATP (Na_2_ATP,3H_2_O; MP Biomedicals; added last to initiate the reaction). Reactions were terminated, and the data analysed, as described previously^38^.

### Lysis assays

Erythrocytes infected with trophozoite stage 3D7 parasites expressing the Hyp1-Nluc fusion protein exported into the erythrocyte compartment were washed in complete RPMI media to remove background levels of the nanoluciferase. To parasitised erythrocytes at 4% haematocrit the following compounds were added: 5 × EC_50_, 10 × EC_50_ and 20 × EC_50_ of MB14 (5 μM, 10 µM and 20 µM, respectively), cipargamin (5 nM, 10 nM and 20 nM, respectively), and artemisinin (0.05 μM, 0.1 µM and 0.2 μM). A DMSO 0.04% v/v vehicle control was also included and triplicate 100 µL samples of the parasitised erythrocytes were added to the wells of 96 well plates. A single 96 well plate was set up for each time point of 0, 1, 2, 4 and 8 h and these were incubated at 37 °C in humidified gas (94% N_2_, 5% CO_2_ and 1% O_2_). At each time point the parasitised erythrocytes were resuspended and 10 μL of suspension was removed from each well into a new plate with 90 μL complete RPMI media and stored at 4 °C until the completion of the time course. At the end of the time course, 10 μL of resuspended culture from each well was aliquoted into the wells of white luminometer plates and 10 μL of 5 × NanoGlo assay buffer containing 1:100 NanoGlo (Promega) and 70 μL RPMI media were added. The plates were immediately read in a luminometer. For the rest of the culture (90 μL), 90 μL of complete parasite media was added and the samples were centrifuged at 250 *g* for 5 min. An 80 µL aliquot of cell free RPMI media was removed to a white luminometer plate and stored at 4 °C until the completion of the time course. At the end of the time course, 10 μL of 5 × NanoGlo assay buffer containing 1:100 NanoGlo (Promega) was added and the plates were immediately read in a luminometer, thereby providing an estimate of the luminescence corresponding to 100% lysis of the infected cells (denoted below as ‘RLU of total culture’). For each time point the corresponding DMSO reading was subtracted to derive the specific degree of lysis induced by the compound treatments.

### Effect of RhopH2 knockdown on the lysis of infected erythrocytes in an isosmotic sorbitol solution

*P. falciparum* RhopH2-HAglmS and 3D7 infected erythrocytes expressing Hyp1-Nluc were treated with 0–1 mM GlcN when at the trophozoite stage (28–36 hours post invasion) and were then grown for a further 48 h until the parasites were trophozoites again. After washing the parasitized erythrocytes twice in PBS, 10 μL of cell suspension (at 1% hematocrit and 1% parasitemia) were dispensed in triplicate into a white 96 well microplate and loaded into a Clariostar luminometer (BMG Labtech). To each well, 40 μL of sorbitol lysis buffer containing the NanoGlo (Promega) substrate (280 mM sorbitol, 20 mM Na-HEPES, 0.1 mg/ml BSA, pH 7.4, Nano-Glo (1:1000 dilution) was added and the relative light units (RLU) measured every 3 min for up to 1 h with gain set to 2500. The RLU increased over time as more cells lysed and released nanoluciferase. The rate of increase in RLU per min (RLU/min) was determined with GraphPad Prism software^52^. A value of 100% lysis is defined as the RLU/min of infected erythrocytes not treated with GlcN (full NPP function) in sorbitol lysis buffer. A value of 0% lysis is defined as the RLU/min of 10 µL parasites in 40 µL of non-lytic PBS containing NanoGlo substrate (1:1000).

### Effect of RhopH2 knockdown on PfATP4-inhibitor-induced lysis of infected erythrocytes

RhopH2-HAglmS trophozoite infected erythrocytes were treated with different doses of GlcN (0 GlcN, 0.25 mM GlcN, 0.5 mM GlcN or 1 mM GlcN) for 48 h to induce knockdown of *RhopH2*. The parasites were washed twice with RPMI medium and lysis assays were set up with 20 x EC_50_ of each of the inhibitors of interest, as above, for time points 0, 2, 4 and 8 h. The 3D7 parasites expressing the Hyp1-Nluc fusion protein exported into the erythrocyte compartment were also set up as a control. For each time point the corresponding reading from cells treated with DMSO (0.04% v/v) alone was subtracted to obtain the degree of lysis induced by the inhibitor treatments. Cell lysis was graphed as RLU of media/RLU of total culture × 100.

### Effect of furosemide on PfATP4-inhibitor-induced lysis of infected erythrocytes

Erythrocytes infected with RhopH2-HAglmS trophozoites were prepared as above, then rinsed in RPMI medium to remove background nanoluciferase. The cells were then incubated with 20 µM MB14, 20 nM cipargamin, 100 nM artemisinin or 0.2% v/v DMSO for 20 min at 37 °C (to allow the inhibitors time to gain entry into the infected cells) before adding the NPP inhibitor, furosemide, at a final concentration of 100 µM. Lysis was assayed at 0, 2, 4 and 8 h after furosemide addition as described above, with the temperature maintained at 37 °C throughout.

### Homology modelling

Protein sequences were obtained from UniProt^65^. The HHpred Server from the Max Plank Institute Bioinformatics Toolkit^66^ was used to detect homologs of each protein. Modeller V9.2^67^ was used to create homology models of each protein. Unstructured regions from each termini were removed. Figures were constructed using the PyMOL Molecular Graphics System, Version 2.0 Schrödinger, LLC.

## Supplementary information

Supplementary Information

## Data Availability

Genomic sequencing data is available from European Nucleotide Archive; accession number PRJEB31982.
