# Supplementary material for: A 4-cyano-3-methylisoquinoline inhibitor of Plasmodium falciparum growth targets the sodium efflux pump PfATP4
Source: Sci Rep. 2019 Jul 16;9:10292. doi: 10.1038/s41598-019-46500-5 (PMC6635429; doi:10.1038/s41598-019-46500-5)
Supplement: Supplementary file 1 — Supplementary Information [file 41598_2019_46500_MOESM1_ESM.pdf]

Supplementary Information

A 4-cyano-3-methylisoquinoline inhibitor of *Plasmodium falciparum* growth targets the sodium efflux pump PfATP4

Paul R. Gilson[1,2*], Rasika Kumarasingha[1], Jennifer Thompson[4], Xinxin Zhang[5], Jocelyn Sietsma Penington[4], Robabeh Kalhor[3], Hayley E. Bullen[1], Adele M. Lehane[5], Madeline G. Dans[1,8], Tania F. de Koning-Ward[8], Jessica K. Holien[7], Tatiana P. Soares da Costa[3], Mark D. Hulett[3], Melissa J. Buskes[3], Brendan S. Crabb[1,2,6], Kiaran Kirk[5], Anthony T. Papenfuss[4,6], Alan F. Cowman[4,6] and Belinda M. Abbott[3].

Affiliations

1. Burnet Institute, Melbourne, Victoria 3004, Australia
2. Monash University, Melbourne, Victoria 3800, Australia
3. La Trobe University, Melbourne, Victoria 3086, Australia
4. The Walter and Eliza Hall Institute of Medical Research, Parkville, Victoria, 3052, Australia.
5. Research School of Biology, Australian National University, Canberra, ACT 2601, Australia
6. University of Melbourne, Melbourne, Victoria 3010, Australia
7. St. Vincent's Institute of Medical Research, Melbourne, Victoria 3065, Australia
8. School of Medicine, Deakin University, Waurn Ponds, Victoria, 3216 Australia,

Contents **Page**

Contents	Page
Table S1	1
Table S2	3
Figure S1	4
Figure S2	5
Figure S3	6
Figure S4	8
Figure S5	9
Figure S6	10
Figure S7	12
Figure S8	13
Figure S9	14
Figure S10	15
Figure S11	16
Figure S12	17

Table S1. Sequence coverage of MB14 resistant *Plasmodium falciparum* parasite genomes and the genomic locations of SNPs.

Sequencing metrics

18 clones sequenced, from parent strain and 4 resistant strains
Samples S1 - S4: parent 3D7 strain
Samples S5, S5v2, S6: resistant strain A
Samples S7-S10: resistant strain B
Samples S11 - S14: resistant strain D
Samples S15 - S18: resistant strain E

**Sample**	**# reads**	**Mean coverage x**
S1, 3D7	50,973,882	335
S2, 3D7	24,269,225	158
S3, 3D7	18,844,221	123
S4, 3D7	24,001,053	157
S5, A	8,139,030	53
S5v2, A	108,310,897	686
S6, A	16,617,257	108
S7, B	13,531,505	88
S8, B	6,424,231	42
S9, B	4,593,504	30
S10, B	67,363,453	441
S11, D	71,122,302	465
S12, D	56,047,755	363
S13, D	19,495,085	126
S14, D	24,593,165	160
S15, E	20,678,547	134
S16, E	15,240,885	99
S17, E	13,773,630	89
S18, E	13,831,193	90

Quality of sequencing was good, and so is depth of coverage.

Structural variations

None of the strains showed large or consistent copy alterations and the only rearrangements detected by GRIDSS were in telomeric regions or erythrocyte membrane genes.

SNVs

Two different small event callers (SNVer and VarScan2) both found SNPs in all resistant clones that were not in the parent 3D7 clones.
One event, Chr 12:531680, was present in all drug-resistant clones. Two more events were present in all strain A samples, but not the other strains. There was also one SNP that was in only 2 of the 4 strain D samples.

Present in strain A samples:

Position	Change	Alternate count / depth
Chr 12:531680	T -> G	82/83, 1091/1096, 102/102
Chr 14:2914869	G -> A	51/51, 861/865, 76/76
Chr 11:751977	A -> C	26/26, 263/263, 81/81

Present in strain B samples:

Position	Change	Alternate count / depth
Chr 12:531680	T -> G	135/135, 49/49, 33/33, 376/377

Present in D samples:

Position	Change	Alternate count / depth
Chr 12:531680	T -> G	289/289, 252/252, 176/176, 184/184
Chr 13:2452844	C -> T	0/289, 256/257, 0/189, 155/156<br>(i.e. present in S12 and S14)

Present in E samples:

Position	Change	Alternate count / depth
Chr 12:531680	T -> G	169/169, 102/103, 118/118, 130/130

SNPs are non-synonymous

Position	Base change	Gene ID	AA change	Gene description
Chr 12:531680	T -> G	PF3D7_1211900	S -> R	non-SERCA-type Ca2+ -transporting P-ATPase
Chr 14:2914869	G -> A	PF3D7_1471200	A -> T	inorganic anion exchanger, SulP
Chr 11:751977	A -> C	PF3D7_1119900	L -> F	protein transport protein Sec16
Chr 13:2452844	C -> T	PF3D7_1361100	P -> L	protein transport protein Sec24A

Table S2. PCR primer sequences used to amplify chromosomal regions covering mutations identified in MB14 resistant parasites via genome sequencing. All SNPs identified form the various resistant lines in Table S1 were present in PCR products.

Name	Sequence 5' to 3'	Size (bp)
ATP4_F	TACCACATCTGTAACTAGCGGTTCTGGT	429
ATP4_R	TATCAGAACATATGACTGAACAGCATCCT	
SulP_F	GTCAGCCTTAGTTTATGTATGGCAAAGCA	537
SulP_R	TATAGTAAAGGCACAACTGGGGCCTCCA	
Sec16_F	TGTGTATATATAATAGTAAGAAAGCTACCAACGT	560
Sec16_R	AGCACTATGAATTTTATTAACGTCCTCTACA	
Sec24A_F	CAAGTCGTTGTATCTTTGTTAGCTCACCA	587
Sec24A_R	ACTGCATAGATCCATTGAGGATTAATGCT	

Figure S1

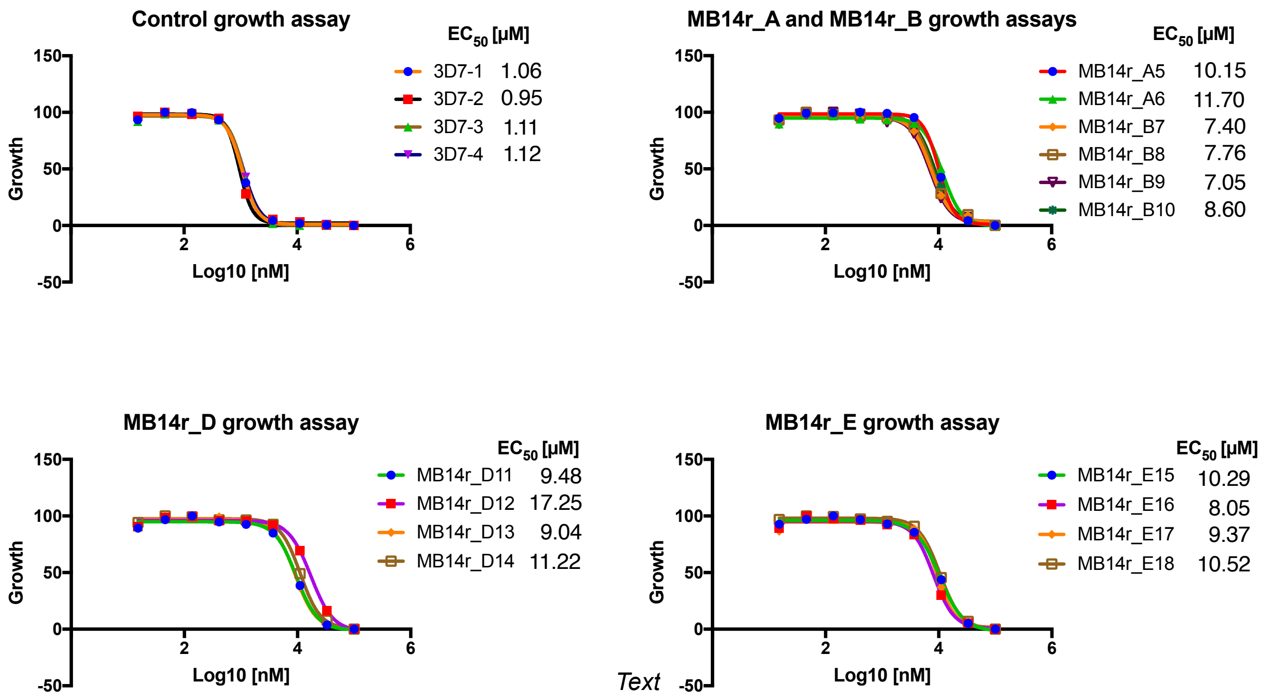

Figure S1. Growth assays of blood stage Plasmodium falciparum 3D7 parental clones versus MB14 resistant clones from populations A, B, D and E. Lactate dehydrogenase activity was measured as a marker of parasite proliferation after a 72 h period of drug treatment. The growth curves were normalised to 100% for full growth.

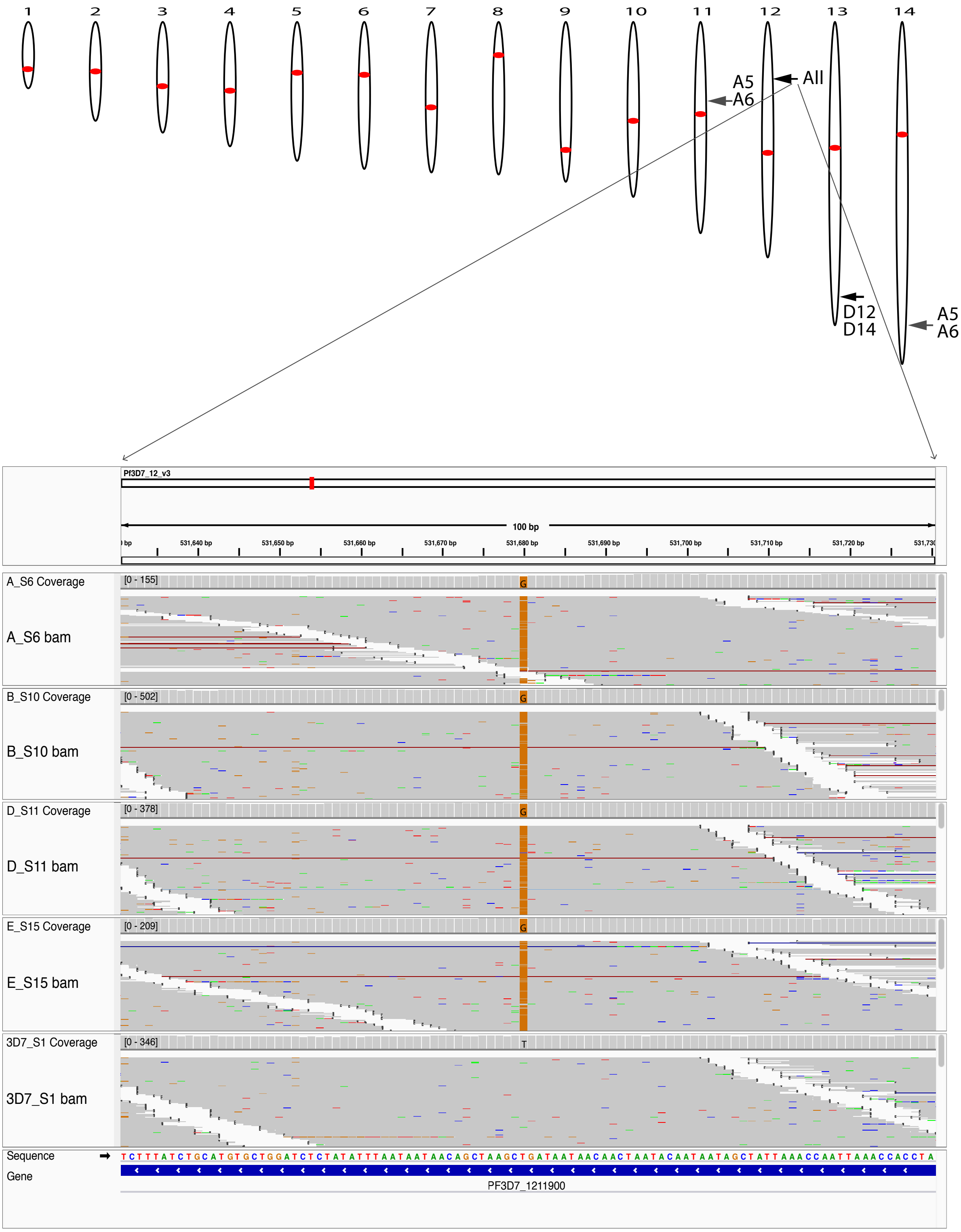

Figure S2. Image from Integrated Genome Viewer showing reads from four MB14 resistant *P. falciparum* parasite strains showing a SNP (T -> G) on chromosome 12 at position 531,680 which is not present in reads from parent 3D7 strain. Alternate allele frequency is 99% - 100% in all samples. The variant is non-synonymous, changing a Serine to an Arginine in a gene for non-SERCA-type Ca2+ -transporting P-ATPase (orange). SNPs unique to strain A (A5 & A6) were also in chromosome 11 and chromosome 14. Another SNP unique to two clones of strain D (D12 & D14) were found on chromosome 13.

Figure S3. CLUSTAL W 2.1 multiple sequence alignment of PfATP4. PfATP4 S374R mutation is highlighted in yellow, transmembrane domains predicted by TMHMM are shaded and drug resistance mutations generated by others are indicated above alignment.

```
WT_ATP4                             MSSQNNNKQGGQDINNKKDSDDIKPSVSKEDLINSLKNDELNKNTTMDQNDMKKNENMNI
ATP4r_R374                          MSSQNNNKQGGQDINNKKDSDDIKPSVSKEDLINSLKNDELNKNTTMDQNDMKKNENMNI
                                    ************************************************************

WT_ATP4                             KKNEVLNNSNNVEDGDNENSKFMNKSKEGLNNINGEKNDDNNSIVKVEESPKSIGYNYYA
ATP4r_R374                          KKNEVLNNSNNVEDGDNENSKFMNKSKEGLNNINGEKNDDNNSIVKVEESPKSIGYNYYA
                                    ************************************************************
                                                                                       H
WT_ATP4                             SESIENLCKEFGLESINTGLNSEQVKINRDKYGENFIEKDEVVPVWLIFLSQYCSPVVLL
ATP4r_R374                          SESIENLCKEFGLESINTGLNSEQVKINRDKYGENFIEKDEVVPVWLIFLSQYCSPVVLL
                                    ************************************************************
                                                                              R
WT_ATP4                             LLVAAVASLALNEVVEGVAIISIVTLNACLATYMEKSSGDAIGKLAEMASPQCTVLRNGQ
ATP4r_R374                          LLVAAVASLALNEVVEGVAIISIVTLNACLATYMEKSSGDAIGKLAEMASPQCTVLRNGQ
                                    ************************************************************

WT_ATP4                             KVVIPSREVVVGDVVLINTGDSISADLRLFDVIELKTNESLLTGESEDIKKTIVADNLST
ATP4r_R374                          KVVIPSREVVVGDVVLINTGDSISADLRLFDVIELKTNESLLTGESEDIKKTIVADNLST
                                    ************************************************************
                                                                                     H    Y  S
WT_ATP4                             PFATNLCFATTSVTSGSGKGIVISTGLDTQVGKIASQLKKSSKGSKLTPLQVALNKLGGL
ATP4r_R374                          PFATNLCFATTSVTSGSGKGIVISTGLDTQVGKIASQLKKSSKGSKLTPLQVALNKLGGL
                                    ************************************************************
                                                                                       L
                                                 R                       F             T  D  N
WT_ATP4                             IGLIAIIVLVVIISLAVIIKYRDPAHADKDPTFVIIIIGVGFAVSSIPEGLPMVVTITLS
ATP4r_R374                          IGLIAIIVLVVIIRLAVIIKYRDPAHADKDPTFVIIIIGVGFAVSSIPEGLPMVVTITLS
                                    ************* **********************************************
                                                    S
WT_ATP4                             AGAKDMVKKNANVRKLPAVETLGCCSVICSDKTGTLTEGKMTAINAVTICKNSSLSDENN
ATP4r_R374                          AGAKDMVKKNANVRKLPAVETLGCCSVICSDKTGTLTEGKMTAINAVTICKNSSLSDENN
                                    ************************************************************

WT_ATP4                             KLTKTFDFYPTKGFEPCGGLFDSNELTSEKKKEIVIAKNQNTSYDKVLYNYGNPSNKSVI
ATP4r_R374                          KLTKTFDFYPTKGFEPCGGLFDSNELTSEKKKEIVIAKNQNTSYDKVLYNYGNPSNKSVI
                                    ************************************************************

WT_ATP4                             VDKTRSLMFAAYLNSYDTTLSRDPKTLKWGIHGNMSEGPIVVAAAKVGYSFINNPNHKSY
ATP4r_R374                          VDKTRSLMFAAYLNSYDTTLSRDPKTLKWGIHGNMSEGPIVVAAAKVGYSFINNPNHKSY
                                    ************************************************************

WT_ATP4                             LDNFQRLDDLEVTFNSSRKMKITFYKLKTVNVFENVYLDKPGKVYTHVALIKGAPDRLLD
ATP4r_R374                          LDNFQRLDDLEVTFNSSRKMKITFYKLKTVNVFENVYLDKPGKVYTHVALIKGAPDRLLD
                                    ************************************************************

WT_ATP4                             RSTHLLEETSMKKVQVSWNSTITQEERNVLIKKNLELSQKALRVLSICIKPLTDQNIEEL
ATP4r_R374                          RSTHLLEETSMKKVQVSWNSTITQEERNVLIKKNLELSQKALRVLSICIKPLTDQNIEEL
                                    ************************************************************

WT_ATP4                             KKLEDADERLKYVNYDENGGFIPMGYVASFDPPRPGVKEAIQTCREAQVKVIMITGDQKP
ATP4r_R374                          KKLEDADERLKYVNYDENGGFIPMGYVASFDPPRPGVKEAIQTCREAQVKVIMITGDQKP
                                    ************************************************************

WT_ATP4                             TAVAIGKLIGLIEEKSEQVEDINSLAIECSELHINKNPNEPILPNDQLDEFTDKILIYSR
ATP4r_R374                          TAVAIGKLIGLIEEKSEQVEDINSLAIECSELHINKNPNEPILPNDQLDEFTDKILIYSR
                                    ************************************************************

WT_ATP4                             AQPEDKITIVQSLKRKGYLVAMTGDGVNDAPALKAADIGVAMGINGTEVAKGASEMILID
ATP4r_R374                          AQPEDKITIVQSLKRKGYLVAMTGDGVNDAPALKAADIGVAMGINGTEVAKGASEMILID
                                    ************************************************************
                                                    L          F
WT_ATP4                             DNFCTVVSAIDVGRTIFSNIQKFVCFLLGTNIGEIIYLSVAIVAQMPFPLEALQILFLNL
ATP4r_R374                          DNFCTVVSAIDVGRTIFSNIQKFVCFLLGTNIGEIIYLSVAIVAQMPFPLEALQILFLNL
                                    ************************************************************
                                         A
                                         T                       R
WT_ATP4                             MTDGCPAVALSREPPNDDNMKTPPRPKKQPIMTKRWWFYGILPHTIFEALCVLLSLAFSL
ATP4r_R374                          MTDGCPAVALSREPPNDDNMKTPPRPKKQPIMTKRWWFYGILPHTIFEALCVLLSLAFSL
                                    ************************************************************
```

```
WT_ATP4              YICTGFYNLNGIHNLCKTVNLVDVNDANVYHEYKYFCSSYEYRISTDYVGWVTNVSFWDP
ATP4r_R374           YICTGFYNLNGIHNLCKTVNLVDVNDANVYHEYKYFCSSYEYRISTDYVGWVTNVSFWDP
                     ************************************************************

WT_ATP4              QNNEAVNFWGAAKGKVENINPLSDIVHPELRLRMQDGCSGDLTLDENGWCRPKDNKTSDG
ATP4r_R374           QNNEAVNFWGAAKGKVENINPLSDIVHPELRLRMQDGCSGDLTLDENGWCRPKDNKTSDG
                     ************************************************************

WT_ATP4              YNDELEGILKKGFEDVTAKGSKRGRTMAFISAVWCEMLRAYTVRSWEPFYKVFNRNMWMH
ATP4r_R374           YNDELEGILKKGFEDVTAKGSKRGRTMAFISAVWCEMLRAYTVRSWEPFYKVFNRNMWMH
                     ************************************************************

WT_ATP4              LACSISATLTFLSTCIPGITSILNTTCLLWWQYLLAIFWALLNLFLDEIVPKVIYRRKYM
ATP4r_R374           LACSISATLTFLSTCIPGITSILNTTCLLWWQYLLAIFWALLNLFLDEIVPKVIYRRKYM
                     ************************************************************

WT_ATP4              TIKN
ATP4r_R374           TIKN
                     ****
```

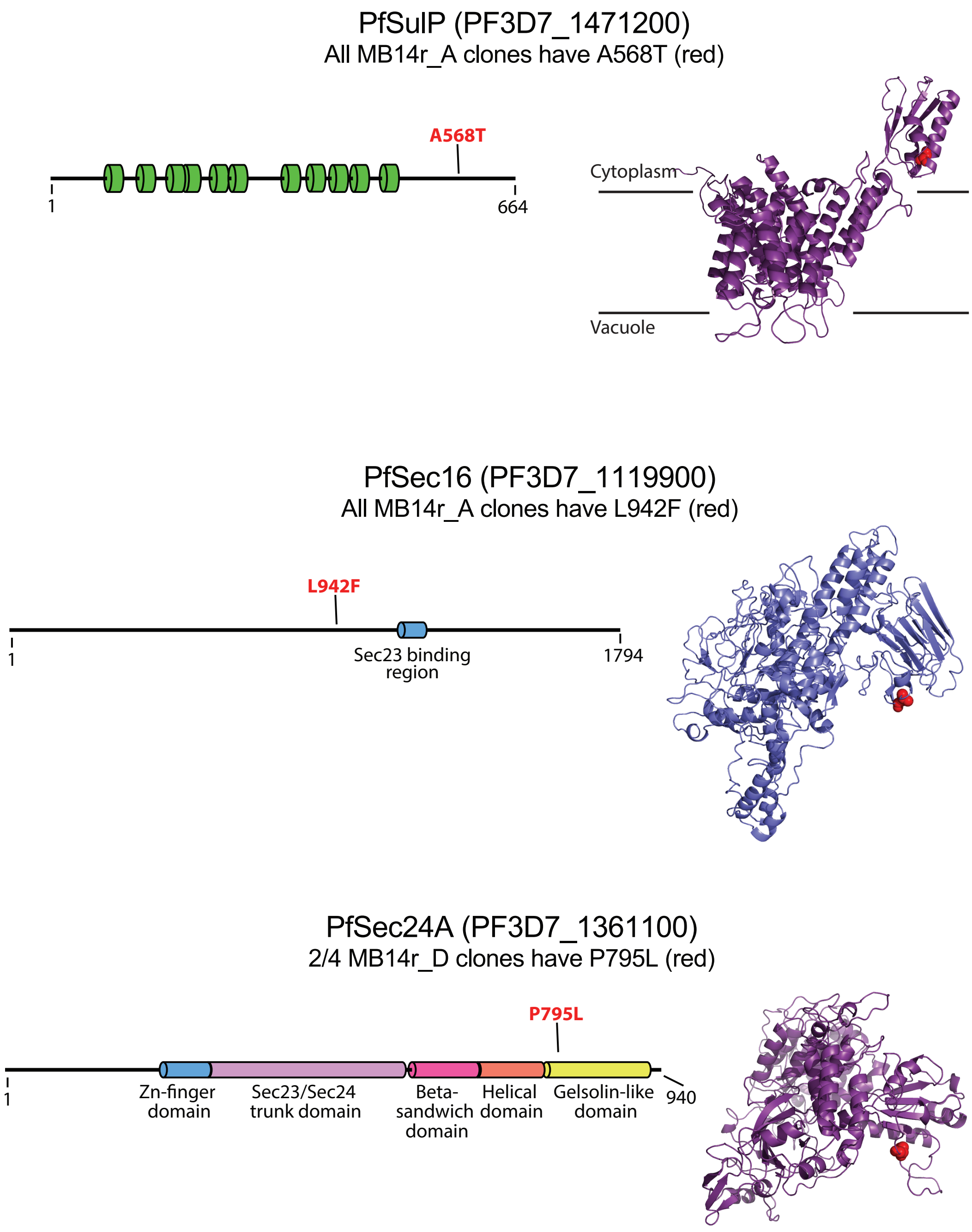

Figure S4. Diagrams and homology models of *Plasmodium falciparum* proteins in which selection for resistance to MB14 produced mutations. Apart from PfATP4, the MB14r_A parasites had mutations in the genes for SulP and Sec16. Wide cyclinders denote predicted transmembrane domains and narrow cylinders indicate conserved protein domains. MB14r_D12 and D14 parasites had an additional mutation in the gene for Sec24. Models were generated by Modeller V9.2 and amino acid mutations are shown in red.

Figure S5. CLUSTAL W 2.1 multiple sequence alignment of SulP with A568T mutation is highlighted in yellow and transmembrane domains are shaded.

```
SulP                                    MLETNKVEDATVNEYVERPEEFNKLDESVERTKLFHNDVYDNDIKICLPTRIGFSKAFMS
SulP_A568T                              MLETNKVEDATVNEYVERPEEFNKLDESVERTKLFHNDVYDNDIKICLPTRIGFSKAFMS
                                        ************************************************************

SulP                                    MVDGIKWGWGFTNTPKETSKYYINEILCGCILCLTMLPEMISFCMIAKIPPYLGLQGASF
SulP_A568T                              MVDGIKWGWGFTNTPKETSKYYINEILCGCILCLTMLPEMISFCMIAKIPPYLGLQGASF
                                        ************************************************************

SulP                                    LSLITSIFGGSPAVIHGVTGAFASVCSKYLVENNNVDLLPDGIERLYICIFICSVMLFFF
SulP_A568T                              LSLITSIFGGSPAVIHGVTGAFASVCSKYLVENNNVDLLPDGIERLYICIFICSVMLFFF
                                        ************************************************************

SulP                                    SYFHMSALIQLIPTPVFIGYCNGLSIIFLRAQLHNLKNPYTHEYIKGHYLLFFIIICTLV
SulP_A568T                              SYFHMSALIQLIPTPVFIGYCNGLSIIFLRAQLHNLKNPYTHEYIKGHYLLFFIIICTLV
                                        ************************************************************

SulP                                    VLIVELWKKIPKVGQKIPSSLIAITVTIFVEFVILRKFLHNNFASFKDVKSFTVGDMFSF
SulP_A568T                              VLIVELWKKIPKVGQKIPSSLIAITVTIFVEFVILRKFLHNNFASFKDVKSFTVGDMFSF
                                        ************************************************************

SulP                                    TSDKAKPTFLFTNKDLNFSKVEFNMDLIKQVANMFTVLLVEALMVSEVIKDMGGAECDTN
SulP_A568T                              TSDKAKPTFLFTNKDLNFSKVEFNMDLIKQVANMFTVLLVEALMVSEVIKDMGGAECDTN
                                        ************************************************************

SulP                                    ETIFSLFIGNVLASLGSAVGGSSLLGLSVLNYRNGARGKESGVVASILIYAILLFGYSLL
SulP_A568T                              ETIFSLFIGNVLASLGSAVGGSSLLGLSVLNYRNGARGKESGVVASILIYAILLFGYSLL
                                        ************************************************************

SulP                                    NYIPLSFLCGIMITVFIHCFKWFSIPIVFFTFCPGYIRNCHPCMSRKISRWDAFIIVLVT
SulP_A568T                              NYIPLSFLCGIMITVFIHCFKWFSIPIVFFTFCPGYIRNCHPCMSRKISRWDAFIIVLVT
                                        ************************************************************

SulP                                    VLCVFVSVPNGVLTGIILSALVYVWQSKSTFKFEIFYDRDTDTKYYEIEGHLFYASKKMF
SulP_A568T                              VLCVFVSVPNGVLTGIILSALVYVWQSKSTFKFEIFYDRDTDTKYYEIEGHLFYASKKMF
                                        ************************************************************

SulP                                    TRLFNYENDSSTVNIVLKGKSTLFDYTAIEALTSVKQQYNVNNKNVTIHGLSHECIKKIA
SulP_A568T                              TRLFNYENDSSTVNIVLKGKSTLFDYTTIEALTSVKQQYNVNNKNVTIHGLSHECIKKIA
                                        ***************************:********************************

SulP                                    KMNHLCKQIDVDLVKVEAPVVPLLYKPLQTIFQKQRTIRRKMSFKIKKKKKEKVEENLND
SulP_A568T                              KMNHLCKQIDVDLVKVEAPVVPLLYKPLQTIFQKQRTIRRKMSFKIKKKKKEKVEENLND
                                        ************************************************************

SulP                                    IEQP
SulP_A568T                              IEQP
                                        ****
```

Figure S6. CLUSTAL W 2.1 multiple sequence alignment of PFSEC16 showing L942F mutation in yellow.

```
PFSEC16                       MKNNLFTYENIFKNKNINIIDKIKEKRKSTQDINNVSIKEKENDNCDVNNKDTQGELSSQ
PFSEC16_L942F_                MKNNLFTYENIFKNKNINIIDKIKEKRKSTQDINNVSIKEKENDNCDVNNKDTQGELSSQ
                              ************************************************************

PFSEC16                       KGNEENIHMDKNNINNKEVCKNDKNIDMCQSLLGKDNLEYTNNNVNGFNNTSEDTFLNFK
PFSEC16_L942F_                KGNEENIHMDKNNINNKEVCKNDKNIDMCQSLLGKDNLEYTNNNVNGFNNTSEDTFLNFK
                              ************************************************************

PFSEC16                       LRKKKNDISSLFDDNTYDMEISYNEKDEKDERNEKDEKDERNEKDEKDERNERNEKDEKD
PFSEC16_L942F_                LRKKKNDISSLFDDNTYDMEISYNEKDEKDERNEKDEKDERNEKDEKDERNERNEKDEKD
                              ************************************************************

PFSEC16                       ERNEKDEKDERNKKNENDEKDKRDDKIFDKNSFFLSPSVVMEPQEEFSSYINSINNNEMN
PFSEC16_L942F_                ERNEKDEKDERNKKNENDEKDKRDDKIFDKNSFFLSPSVVMEPQEEFSSYINSINNNEMN
                              ************************************************************

PFSEC16                       NNNNNNNNSSSNYRNNIFYDNVETFNVINETQNEGNENLHIIKKGKVENYFNDCKKEFLV
PFSEC16_L942F_                NNNNNNNNSSSNYRNNIFYDNVETFNVINETQNEGNENLHIIKKGKVENYFNDCKKEFLV
                              ************************************************************

PFSEC16                       DKNFNDEYYEEDINNYMSNNKVEKLIIDKEKENNEYNKINGDNFHHDNNINITEHLNNDS
PFSEC16_L942F_                DKNFNDEYYEEDINNYMSNNKVEKLIIDKEKENNEYNKINGDNFHHDNNINITEHLNNDS
                              ************************************************************

PFSEC16                       LIIYSHKTYFHDPNLNDTNSQHINNENVILKEKGEEENESNNNQKDYNKIRESVSLNNNI
PFSEC16_L942F_                LIIYSHKTYFHDPNLNDTNSQHINNENVILKEKGEEENESNNNQKDYNKIRESVSLNNNI
                              ************************************************************

PFSEC16                       MDNNSFLWNNKNDRMSCEYNEDRNICSENLKDIFNNEQEKEYMKMNIHNNNNNNNKYIYD
PFSEC16_L942F_                MDNNSFLWNNKNDRMSCEYNEDRNICSENLKDIFNNEQEKEYMKMNIHNNNNNNNKYIYD
                              ************************************************************

PFSEC16                       NINLYHKGDNVHMNVATIKDHNNNVDVISKKDHINIPSVHKNVHVSFCFGNNGTFFYSKN
PFSEC16_L942F_                NINLYHKGDNVHMNVATIKDHNNNVDVISKKDHINIPSVHKNVHVSFCFGNNGTFFYSKN
                              ************************************************************

PFSEC16                       SKIKYQSLINVIEIHINKKKRKMHIFNEKNNEENNIYMNSNCNYNNYCNRNVDSNDVKHC
PFSEC16_L942F_                SKIKYQSLINVIEIHINKKKRKMHIFNEKNNEENNIYMNSNCNYNNYCNRNVDSNDVKHC
                              ************************************************************

PFSEC16                       YHNNMEKFIYCIKNFPGPFSRKSNKVDHKIEEFLKGYININKSRSVNDNIFEDIQKNCLY
PFSEC16_L942F_                YHNNMEKFIYCIKNFPGPFSRKSNKVDHKIEEFLKGYININKSRSVNDNIFEDIQKNCLY
                              ************************************************************

PFSEC16                       NFLLNILKKPYLTNFNLKEISIEKKNDINKNMNHKGNNIISYFLDNYQEKKDIKDDIYLS
PFSEC16_L942F_                NFLLNILKKPYLTNFNLKEISIEKKNDINKNMNHKGNNIISYFLDNYQEKKDIKDDIYLS
                              ************************************************************

PFSEC16                       DDCNEDEDIDIDSLSNNEMDEFDINEYVKNDSNKMKKYDEPCNNNHINIYNPTNNNSNNI
PFSEC16_L942F_                DDCNEDEDIDIDSLSNNEMDEFDINEYVKNDSNKMKKYDEPCNNNHINIYNPTNNNSNNI
                              ************************************************************

PFSEC16                       IMNNNVDKKKNVLNKDQKKCYTDVYENSKNINDICINNNTYFFFDFINKEFIMELYKLEN
PFSEC16_L942F_                IMNNNVDKKKNVLNKDQKKCYTDVYENSKNINDICINNNTYFFFDFINKEFIMELYKLEN
                              ************************************************************

PFSEC16                       KNERITHDDIYLLYFHMCIYNSKKATNVCIKKELYKYFFLVLRKYNKKKYYKMLDKYISY
PFSEC16_L942F_                KNERITHDDIYLLYFHMCIYNSKKATNVCIKKELYKYFFLVLRKYNKKKYYKMLDKYISY
                              ************************************************************

PFSEC16                       IKRSINNELEMKNDMKNIYKIYNYNIYDEICTEAFVFFLCILNKKNMVFKKNILVNYWYA
PFSEC16_L942F_                IKRSINNELEMKNDMKNIYKIYNYNIYDEICTEAFVFFLCIFNKKNMVFKKNILVNYWYA
                              *****************************************:******************

PFSEC16                       FYTLIFHNFIFQYKENEIQFADYKEDIINFFIYLIHLLYEKKKNAEAQFLLLLISNNPFV
PFSEC16_L942F_                FYTLIFHNFIFQYKENEIQFADYKEDIINFFIYLIHLLYEKKKNAEAQFLLLLISNNPFV
                              ************************************************************

PFSEC16                       FSFSPNTKNDDNYVEDVNKIHSANIGSNQMYSDNIYGNNIHSNNNMRGNNIYCNNNHPSH
PFSEC16_L942F_                FSFSPNTKNDDNYVEDVNKIHSANIGSNQMYSDNIYGNNIHSNNNMRGNNIYCNNNHPSH
```

```
                                  ************************************************************

PFSEC16                           SNNYIRSNNYIRSNNYIHGNNYIRSNDLYNRSNIEESNAVGYCGLLNSHFDIMCFQVCDI
PFSEC16_L942F_                    SNNYIRSNNYIRSNNYIHGNNYIRSNDLYNRSNIEESNAVGYCGLLNSHFDIMCFQVCDI
                                  ************************************************************

PFSEC16                           YEYMCRYEKDDFFFEQLIFYKIIYAYILLEYGILSQAQRYVEILYYYIDVIKNDRKKNSY
PFSEC16_L942F_                    YEYMCRYEKDDFFFEQLIFYKIIYAYILLEYGILSQAQRYVEILYYYIDVIKNDRKKNSY
                                  ************************************************************

PFSEC16                           LLYLYDTLLEKTKYIFTTTTNKDMLSLQRKSCMSNDEVIYTYKFISNSGITTTIPKDNMY
PFSEC16_L942F_                    LLYLYDTLLEKTKYIFTTTTNKDMLSLQRKSCMSNDEVIYTYKFISNSGITTTIPKDNMY
                                  ************************************************************

PFSEC16                           EHMMDKNHSNNIKGYVNYNNMNDTNVNYINEYTTTNTNINVFNNSPIHNRNKTIHVQTQE
PFSEC16_L942F_                    EHMMDKNHSNNIKGYVNYNNMNDTNVNYINEYTTTNTNINVFNNSPIHNRNKTIHVQTQE
                                  ************************************************************

PFSEC16                           NINEYFNIKTNRTVTDHLGKNNVYSNIKEEKLNSTMFDILKSNNNNSSYNENENIQSCDM
PFSEC16_L942F_                    NINEYFNIKTNRTVTDHLGKNNVYSNIKEEKLNSTMFDILKSNNNNSSYNENENIQSCDM
                                  ************************************************************

PFSEC16                           LNGDNHTNRNEDSSIIHIQNGLSVHQAINNNNNNIYHNNNNIYPNNNNMYHSNNNMYHSN
PFSEC16_L942F_                    LNGDNHTNRNEDSSIIHIQNGLSVHQAINNNNNNIYHNNNNIYPNNNNMYHSNNNMYHSN
                                  ************************************************************

PFSEC16                           NNNNMYYNNNKYYTYEHASSNVNPSSYFAAEQNEAPYNFTINNKNNNIGTNYNFPSVNNN
PFSEC16_L942F_                    NNNNMYYNNNKYYTYEHASSNVNPSSYFAAEQNEAPYNFTINNKNNNIGTNYNFPSVNNN
                                  ************************************************************

PFSEC16                           THGHMYNDINNHVNTYEKSTEGTYRYNVPNNDNNNNNNNNITFVYSQNFTNVVEQNNNAK
PFSEC16_L942F_                    THGHMYNDINNHVNTYEKSTEGTYRYNVPNNDNNNNNNNNITFVYSQNFTNVVEQNNNAK
                                  ************************************************************

PFSEC16                           DTVQVQNEKGTMCGPLNVHINTHNSLYDNKNVEGNFTNNIKKDNNMNVKNNNIHINNVNV
PFSEC16_L942F_                    DTVQVQNEKGTMCGPLNVHINTHNSLYDNKNVEGNFTNNIKKDNNMNVKNNNIHINNVNV
                                  ************************************************************

PFSEC16                           QNSSDNNLSSSQDQQQCANENNMDLINMGKSFISGFFSNIKEKIKKTEYMQEEEEEEENI
PFSEC16_L942F_                    QNSSDNNLSSSQDQQQCANENNMDLINMGKSFISGFFSNIKEKIKKTEYMQEEEEEEENI
                                  ************************************************************

PFSEC16                           FYYDYEKKRWREKGVTSDEEKEREKQKLEKQMAMKNISPPPTGINYSSERNKNPLNMTDV
PFSEC16_L942F_                    FYYDYEKKRWREKGVTSDEEKEREKQKLEKQMAMKNISPPPTGINYSSERNKNPLNMTDV
                                  ************************************************************

PFSEC16                           RSRYVDYFN
PFSEC16_L942F_                    RSRYVDYFN
                                  *********
```

Figure S7. CLUSTAL W 2.1 multiple sequence alignment Sec24A with P795L mutation shown in yellow.

```
SEC24A                        MQPYDYNRGLNNNNTANYNNQNNANAPINNPFLHNNDIGNNNNMKTNEGPYNAPTYYNPG
SEC24A_P795L_                 MQPYDYNRGLNNNNTANYNNQNNANAPINNPFLHNNDIGNNNNMKTNEGPYNAPTYYNPG
                              ************************************************************

SEC24A                        QHQQQQQQQQQQGPPLHQGYQHSGVYGMNQGNYKTNNIGENMYNQDGHNNTSYINQGQPY
SEC24A_P795L_                 QHQQQQQQQQQQGPPLHQGYQHSGVYGMNQGNYKTNNIGENMYNQDGHNNTSYINQGQPY
                              ************************************************************

SEC24A                        RNVTSQFIPVSSNNTLKAGGNMLGYDNMGNINHVQPIINDTYQEFLQFNAFSHFVKSSVS
SEC24A_P795L_                 RNVTSQFIPVSSNNTLKAGGNMLGYDNMGNINHVQPIINDTYQEFLQFNAFSHFVKSSVS
                              ************************************************************

SEC24A                        YMPANTTLKQKAYVPLGFVIQPLAPIPDGYPELASVNFGNSTVVRCKKCRTYINPFVRFE
SEC24A_P795L_                 YMPANTTLKQKAYVPLGFVIQPLAPIPDGYPELASVNFGNSTVVRCKKCRTYINPFVRFE
                              ************************************************************

SEC24A                        AGGKKWNCNMCYNINDTPQFYFVPLDEKGKRKDLFQRPELCTGSVEFIAPSDYMIRPPQP
SEC24A_P795L_                 AGGKKWNCNMCYNINDTPQFYFVPLDEKGKRKDLFQRPELCTGSVEFIAPSDYMIRPPQP
                              ************************************************************

SEC24A                        SVYLFLIDVTVTSVNSGLLDVVCSTIKSLLPKNNDSTENNNQDNNNNNNNNNSKNLKSF
SEC24A_P795L_                 SVYLFLIDVTVTSVNSGLLDVVCSTIKSLLPKNNDSTENNNQDNNNNNNNNNSKNLKSF
                              ************************************************************

SEC24A                        DSRTLIGIMTFDSTIHFYNLNSNLKQTQMMVVPDIQDIFIPLPEDILVNVHECQNVIDVL
SEC24A_P795L_                 DSRTLIGIMTFDSTIHFYNLNSNLKQTQMMVVPDIQDIFIPLPEDILVNVHECQNVIDVL
                              ************************************************************

SEC24A                        LDNLPGMWRNNKISDCCAGNALKAAFMVLKKVGGKLLFFLSSVPNIGDLTVSVNRDNKDK
SEC24A_P795L_                 LDNLPGMWRNNKISDCCAGNALKAAFMVLKKVGGKLLFFLSSVPNIGDLTVSVNRDNKDK
                              ************************************************************

SEC24A                        SKYKNIYSSSSSGNNVVDSKLREVELLNPCNNLYAELAQNITQYQIAVDLFACPLYNLDL
SEC24A_P795L_                 SKYKNIYSSSSSGNNVVDSKLREVELLNPCNNLYAELAQNITQYQIAVDLFACPLYNLDL
                              ************************************************************

SEC24A                        ASIYPLIKNSGGSLYYYPQFNVHQYNDKLRQELLFALTTETAWESVMRIRISRGWKITNW
SEC24A_P795L_                 ASIYPLIKNSGGSLYYYPQFNVHQYNDKLRQELLFALTTETAWESVMRIRISRGWKITNW
                              ************************************************************

SEC24A                        YGNYQFRGADLLALPNCHSGQNFSIIVDLEENVVQDSIVYVQSALLYTNSNGERRIRLHT
SEC24A_P795L_                 YGNYQFRGADLLALPNCHSGQNFSIIVDLEENVVQDSIVYVQSALLYTNSNGERRIRLHT
                              ************************************************************

SEC24A                        YALPITQNIKTITDSINPQVVVSLLAHQSIDISKKGKIADGRNLIQNLCSQVLSSQLLQS
SEC24A_P795L_                 YALPITQNIKTITDSINPQVVVSLLAHQSIDISKKGKIADGRNLIQNLCSQVLSSQLLQS
                              ************************************************************

SEC24A                        ECARLLSLYILGMLKSIAFRDSGDVPPDLRIYHWYRLENIPVESVEANFYPRMFSLHNLE
SEC24A_P795L_                 ECARLLSLYILGMLKSIAFRDSGDVPPDLRIYHWYRLENIPVESVEANFYPRMFSLHNLE
                              ************************************************************

SEC24A                        KHHGHLDENNNIVFPDALNLTCENMTQDGCYIVEDGETIVMWIGRSINPQWIYAVFGVQT
SEC24A_P795L_                 KHHGHLDENNNIVFLDALNLTCENMTQDGCYIVEDGETIVMWIGRSINPQWIYAVFGVQT
                              ************** *********************************************

SEC24A                        IDQLNTEYAENHLGSTGNPFGVQILNIINALRKIRTPCYMKLLVVKQGDPLEYKFFSYLI
SEC24A_P795L_                 IDQLNTEYAENHLGSTGNPFGVQILNIINALRKIRTPCYMKLLVVKQGDPLEYKFFSYLI
                              ************************************************************

SEC24A                        EDRSQHMMLSLKEFLAKICPKFPQFTPSMTTNPLATQHGR
SEC24A_P795L_                 EDRSQHMMLSLKEFLAKICPKFPQFTPSMTTNPLATQHGR
                              ****************************************
```

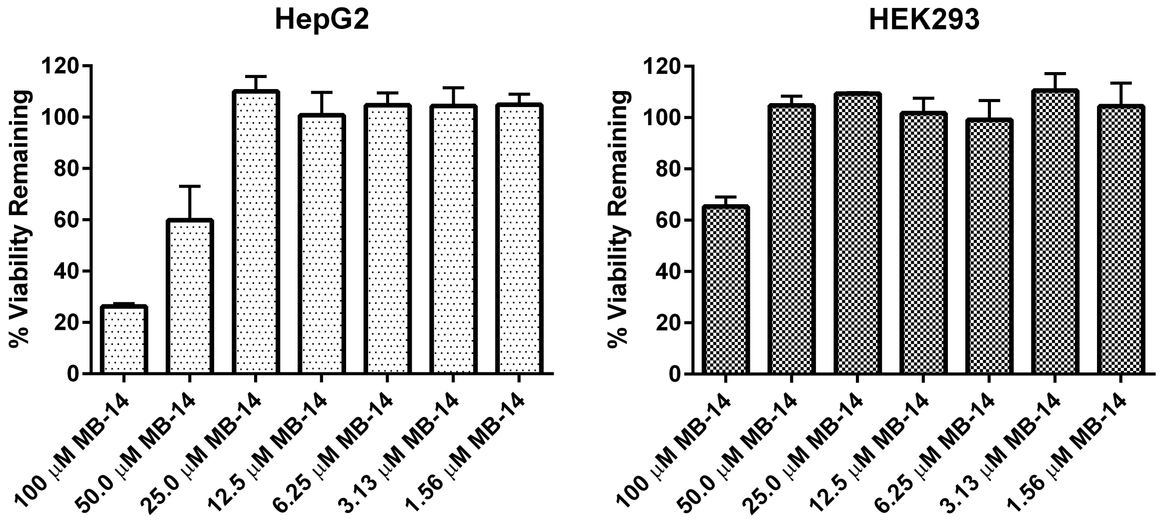

Figure S8. MB14 only reduces the viability of human cells lines at the highest concentrations assayed. Human HepG2 and HEK293 cells lines were treated with the concentrations of MB14 indicated above in 2.5% DMSO for 48 h. Cell proliferation was assayed by measuring the activity of NAD(P)H-dependent cellular oxidoreductase enzymes. Bars indicate mean and error bars SD of 3 technical replicates.

Figure S9

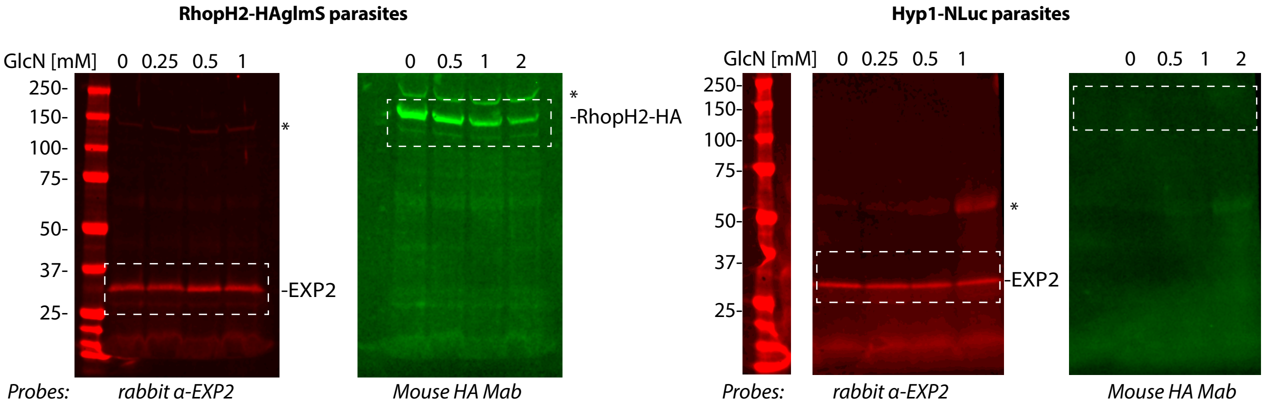

Figure S9. Western blots showing glucoasamine dependent knockdown of RhopH2-HA in RhopH2-HAglmS parasites. Both RhopH2-HAglmS and control Hyp1-Nluc parasites were treated with the concentrations of glucoasmine (GlcN) indicated on top of the blots for 48 h. Trophozoite stage infected eyrthocytes were saponin lysed to remove hemoglobin and were fractionated by SDS-PAGE and transferred to nitrocellulose membranes. Both RhopH2-HA and Hyp1-Nluc membranes were probed with rabbit anti-EXP2 IgG at 5 µg/mL and mouse monoclonal for hemagglutinin at 1 µg/mL. These were then detected with goat anti-rabbit IgG-680nm and goat anti-mouse IgG-800nm and the blots were imaged with an Odyssey Fc scanner. The blot images from each fluorescent channel are shown side by side. Precision Plus Protein All Blue Prestained Protein markers are shown on the left and their sizes indicated in kilobases. Note, the protein markers from the Hyp1-Nluc blot are shown separately because irrelevent samples next to the markers were removed. Asterisks indicate non-specific protein bands. The regions of these blots used to construct Figure 5 are indicated by white dashed boxes.

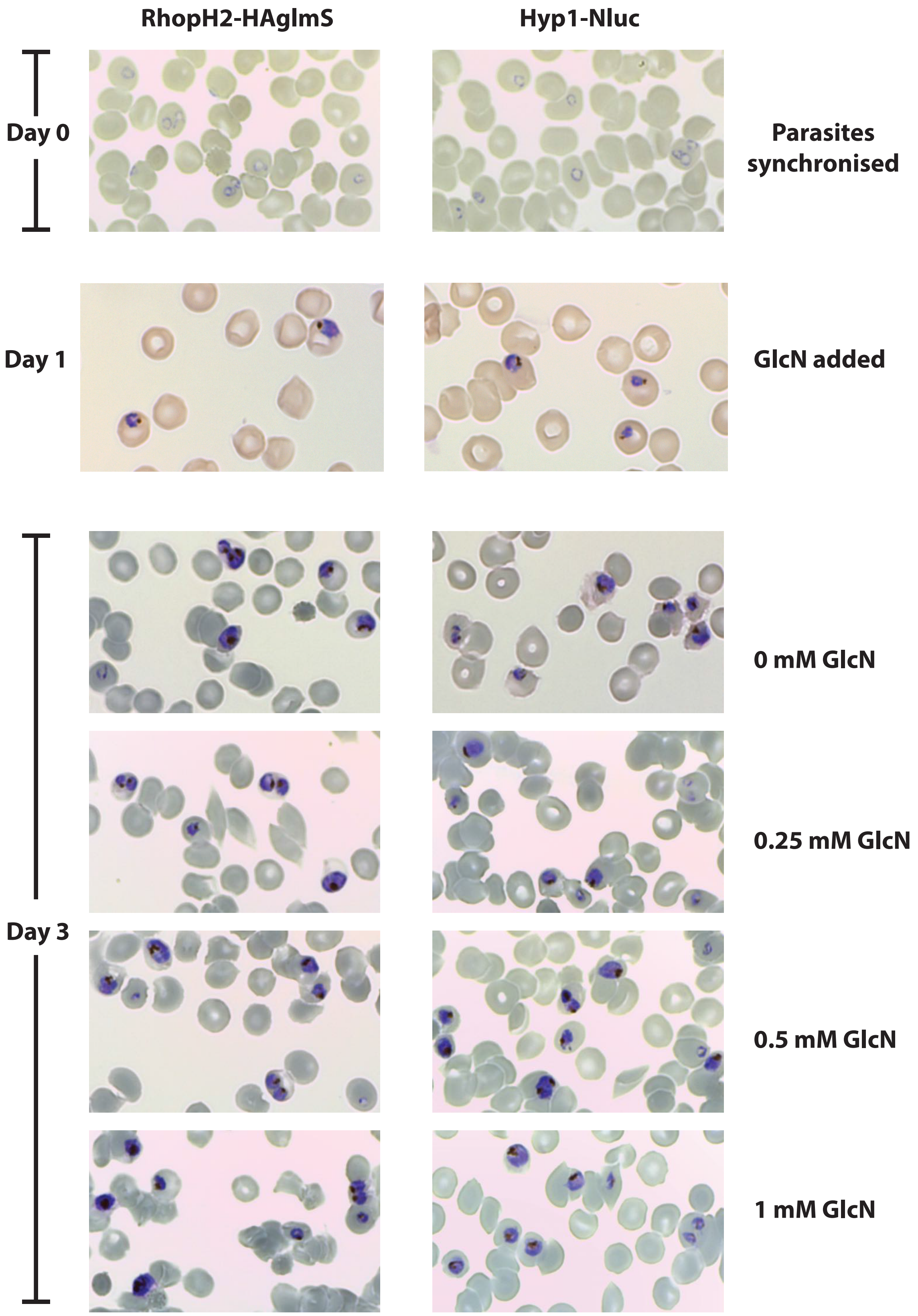

Figure S10. Giemsa stained blood smears of *P. falciparum* parasites before and after GlcN treatment indicates similar developmental stages were assessed in PfATP4 inhibitor lysis assays. RhopH2-HAglmS and Hyp1-Nluc ring stage parasites were sorbitol sychronised on day 0. On day 1they were treated with various GlcN concentrations for ~48 h. On day 3 images of these parasites indicated they had grown to similar sized trophozoites and that GlcN treatment had not hindered development prior to the 8 h PfATP4 inhibitor lysis assay.

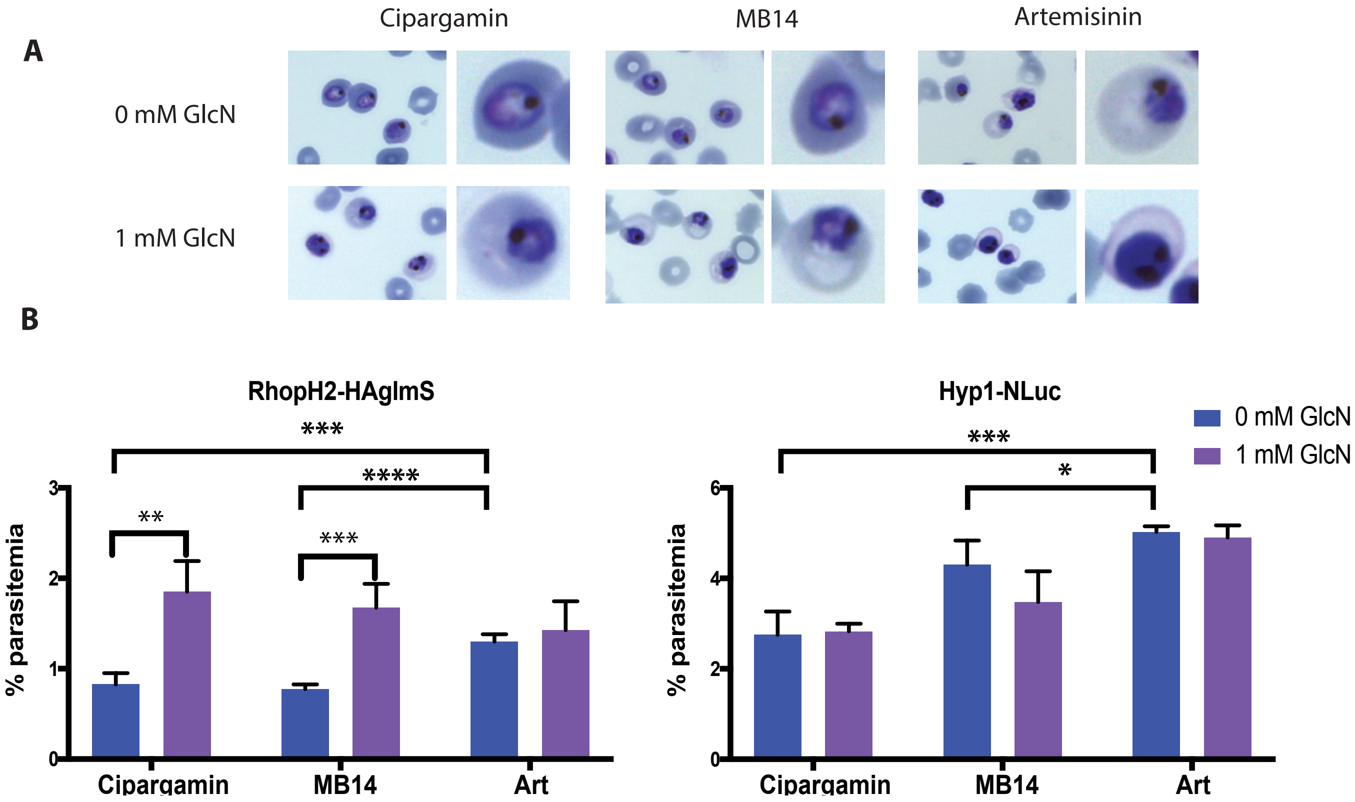

Figure S11. Giemsa stained blood smears of *P. falciparum* indicate PfATP4 inhibitors cipargamin and MB14 may swell and lyse the parasites thereby reducing % parasitemia. **A.** Giemsa stained *P. falciparum* cells develop a light staining centre after 8 hours treatment with cipargamin (10 nM) and MB14 (10 µM) but not artemisnin (100 nM) that could be indicative of cell swelling. The 'swollen' appearance is reduced in cipargamin and MB14 following the knockdown of RhopH2-HA with 1mM GlcN. **B.** Blood smear counts of RhopH2-HAglmS parasites showed that the knockdown of RhopH2-HA with 1 mM GlcN reduced the pro-lytic effect of 8 h treatment with cipargamin and MB14. In a control experiment with Hyp1-Nluc parasites, GlcN treatment had no effect upon lysis. The graph bars indicate mean parasitemias of 4 x 1000 erythrocyte counts. Error bars indicate SED, *p<0.05, **p<0.01, paired T-test. Differences that are not significant are not indicated.

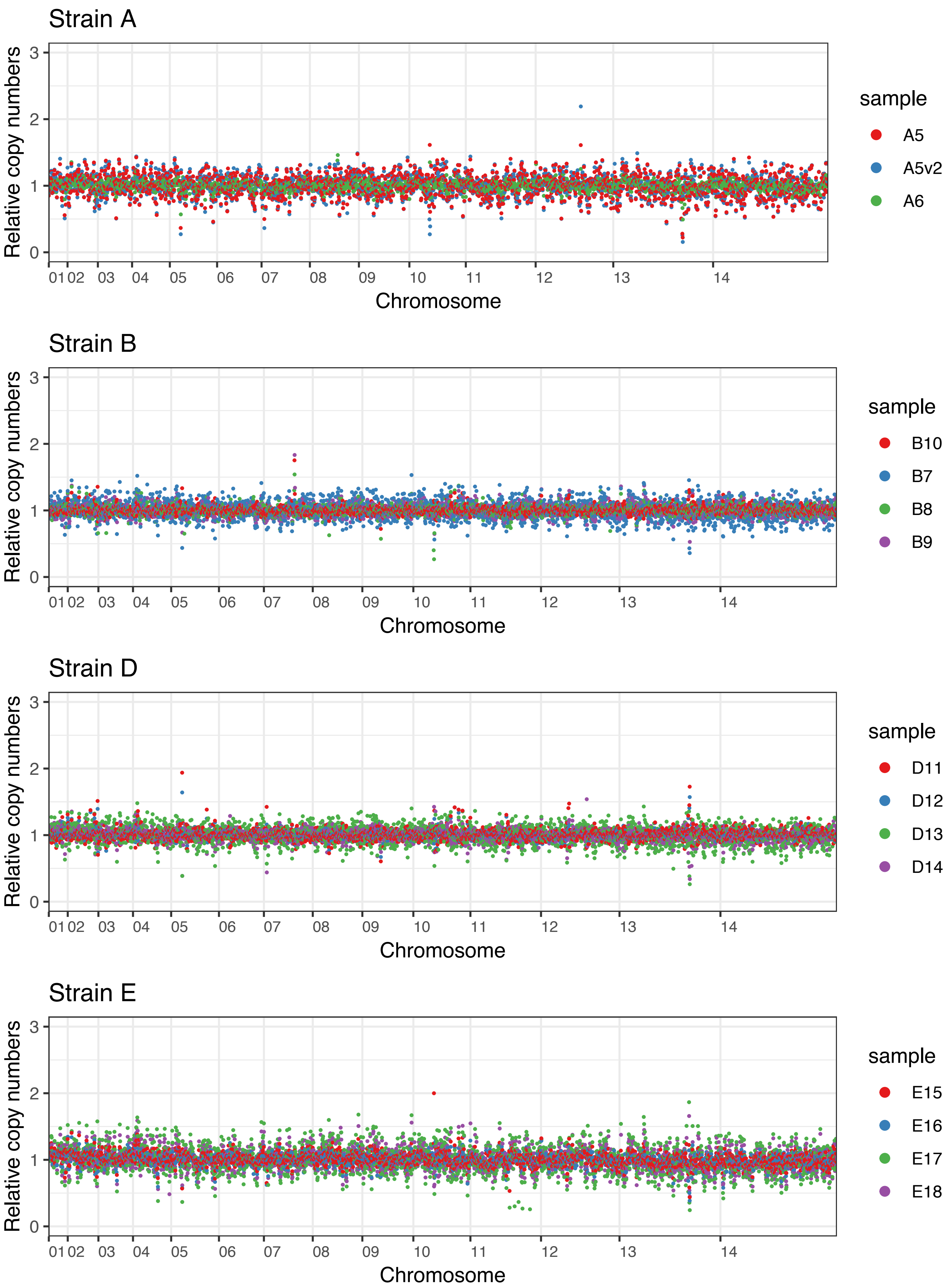

Figure S12. Relative copy numbers of MB14 resistant *Plasmodium falciparum* blood stage parasites. The copy numbers of 10 kb chromosomal regions (bins) from clones of four MB14 resistant populations are shown relative to the parental 3D7 control line. There are no common amplifications or deletions. The small variation around chr13:1.5Mbp is an artifact of a deletion in the parent strain.
